# Defense response changes in roots of oil palm (*Elaeis guineensis* Jacq.) seedlings after internal symptoms of *Ganoderma boninense* Pat. infection

**DOI:** 10.1186/s12870-022-03493-0

**Published:** 2022-03-24

**Authors:** Rokhana Faizah, Riza Arief Putranto, Vivi Restu Raharti, Nanang Supena, Dewi Sukma, Asmini Budiani, Sri Wening, Sudarsono Sudarsono

**Affiliations:** 1 Plant Breeding and Biotechnology Study Program, Department of Agronomy and Horticulture, Faculty of Agriculture, Bogor Agricultural University (IPB University), Jl. Meranti, Dramaga Campus, Bogor, 16680 Indonesia; 2 Indonesian Oil Palm Research Institute, Jl. Brigjen Katamso No. 51, Medan, North Sumatera 20158 Indonesia; 3 Indonesian Research Institute for Biotechnology and Bioindustry, Jl. Taman Kencana No. 1, Bogor, 16128 Indonesia; 4 PT Riset Perkebunan Nusantara (Nusantara Estate Crops Research), Jl. Salak no. 1A, Bogor, 16128 Indonesia; 5 Department of Agrotechnology, Agriculture Faculty, Jenderal Soedirman University, Jl. Dr. Soeparno No. 63, Karangwangkal, North Purwokerto, Central Java 53122 Indonesia

**Keywords:** Defense response, Lignin of oil palm root, Salicylic acid; peroxidase enzyme, Relative expression genes, Basal stem rot disease

## Abstract

**Background:**

The development of basal stem rot (BSR) disease in oil palm is associated with lignin during vegetative growth and salicylic acid (SA) biosynthesis. The increase in the lignin content, SA accumulation, growth, and root biomass could indicate the resistance of oil palm seedlings to BSR disease. Therefore, although there are many studies on the interactions between the *Ganoderma boninense* and oil palm, research on evaluation of physiological processes, biochemistry, and molecules occurring during early internal symptoms of BSR in roots of oil palm (*Elaeis guineensis* Jacq.) are essential.

**Results:**

*Ganoderma boninense* inoculation indicated that C01, C02, and C05 seedlings were susceptible, while the other three seedlings, C03, C07, and C08, were resistant based on *Ganoderma* Disease Index (GDI). Infection by *G. boninense* in the most susceptible seedlings C05 reduced fresh weight of roots (FW) by 9.0%, and lignin content by 10.9%. The most resistant seedlings C08 were reduced by only 8.4%, and 0.2% regarding their fresh weight and lignin content, respectively. BSR disease induced SA accumulation in the most susceptible C08 and decreased peroxidase (PRX) enzyme (EC 1.11.1.7) activities in root tissues of oil palm seedlings except C07 and C08 where PRX activities remained high in the 4 months after planting. Infection with *G. boninense* also increased glutathione S-transferase U19-like (EgGSTU19) gene expression in the root tissues of susceptible seedlings, while laccase-24 (EgLCC24) gene expression was associated with resistance against BSR disease. Based on the relative expression of twelve genes, two genes are categorized as receptors (EgWAKL5, EgMIK1), two genes as biosynthesis signal transduction compound (EgOPR5, EgACO1), five genes as defense responses (EgROMT, EgSOT12, EgLCC24, EgGLT3, EgGSTU19), and one gene as trans-resveratrol di-O-methyltransferase-like (EgRNaseIII) predicted related to BSR infection. While two other genes remain unknown (EgUnk1, EgUnk2).

**Conclusions:**

*Ganoderma* infection-induced SA accumulation and lignification in resistant accessions promote the seedlings root biomass. Oil palm seedlings have a synergistic physical, biochemical, and molecular defense mechanism to the BSR disease. The utilization of nucleotide-based molecular markers using EgLCC24 gene is able to detect resistant oil palm seedlings to *G. boninense*.

**Supplementary Information:**

The online version contains supplementary material available at 10.1186/s12870-022-03493-0.

## Background

Oil palm breeders have focused their breeding programs on developing high-yielding oil palm varieties for effective and efficient cultivation [1, 2]. However, a recent outbreak of basal stem rot (BSR) disease caused by infection from *Ganoderma boninense* Pat. has shifted part of the oil palm breeding focus in a different direction [3, 4]. A significant reduction in oil palm yield may occur because of *G. boninense* in the soil or the planting of materials infected with the pathogen [5, 6]. If the oil palm plants are not infected by *G. boninense* and there are no other pests or diseases, its yearly yield remains high. High oil palm yield occurred from 1998 to 2008. There was an average increase of 4% per year in oil palm [7]. In the endemic areas, *G. boninense* could infect all stages of oil palm development and reduce their yield. Therefore, developing a BSR disease-resistant oil palm varieties has become a significant endeavor in some oil palm breeding programs in Indonesia and other parts of the worldwide [4, 8]. The abiotic stresses such as drought and temperature increase 1 °C are able to reduce the average palm oil productivity by up to 40% [9]. The effects of La Niña and El Niño's stress also decrease the production of crude palm oil (CPO) to 3.37% and the availability of CPO stocks up to 2.5% [10].

Basal stem rot disease is one of at least 16 known diseases infecting oil palm at different tree growth and developmental stages [11]. The BSR has spread rapidly in endemic North Sumatra areas and some regions in Indonesia, where oil palm plantations have been thriving for many generations[12, 13]. *G. boninense* is a soil-borne pathogen [14, 15]. The oil palm roots are essential in *G. boninense* infection [6, 14]. The susceptibility and resistance of plants to BSR disease infection depends on the ability of oil palm roots to interact with the source of the inoculum pathogens of BSR disease [16, 17]. The symptoms of *G. boninense* infection in oil palm roots mainly includes the presence of root necrosis [18, 19]. Other symptoms include *Ganoderma* fruiting body formation at the basal stem and chlorosis with necrotic tips in young, unfolded leaves [2, 20].

Plants are in their most vulnerable phase when they are least able to activate their defense mechanisms [21, 22]. The most critical occurs when the oil palm seedlings are transplanted into soil infested with *G. boninense*. Hence, the Indonesian Oil Palm Research Institute (IOPRI) has developed a standard resistance evaluation method for oil palm seedlings against BSR disease [23]. The IOPRI screening oil palm germplasm using the developed method identified some oil palm accessions carrying resistance mechanisms against *G. boninense* [23]. In different studies, several oil palm provenances with field resistance characteristics have also been identified in BSR disease-infested plantations using the *Ganoderma* infection index [13, 24, 25]. The study also showed that the genetic background from Deli *dura* is more susceptible against *G. boninense* to African *tenera/pisifera* such as La Mé, AVROS, and Yangambi populations [13, 17].

Alleviating the negative impacts of *Ganoderma* infection may be accomplished through different approaches, such as increasing plant vigor by adopting the proper composition of fertilizers [26], bio fungicide application [27], endophytic microbe deployment [14, 28, 29], and spraying fungicides. Unfortunately, these treatments are not always adequate in controlling the disease and requires comprehensive agronomic interventions [8] to contain the spread of BSR disease. The practical measure for preventing BSR disease infestation is to plant oil palm *G. boninense-*resistant donor parents [4, 30].

Breeding for BSR disease-resistant oil palm varieties need resistant donor parents [30]. The desired donors with BSR disease resistance probably have specific physiological, biochemical, and molecular characteristics when interacting with *G. boninense* [31–33]. For example, lignin deposition in root tissues [15, 34, 35] and other vegetative parts [34] are attributes associated with resistance responses. Moreover, peroxidase (PRX) activities [26, 36] and salicylic acid (SA) content [37, 38] have also been suggested as active defense mechanisms against *G. boninense* infection.

The activity of gene expression in oil palm that responds to *G. boninense* infection is equivalently critical. Several genes have been identified with their biological functions. To illustrate, the cytosolic sulfotransferase 12-like gene (AtSOT12) is implicated in sulfonate SA in *A. thaliana* [39, 40]. The glutathione S-transferase U19-like gene (AtGSTU19) is involved in peroxidase activity and is associated with cytosolic [41]. Another gene is putative 12-oxophytodienoate reductase 5 (OsOPR5), which is active in rice roots against the rice root-knot nematode *Meloidogyne graminicola* [42]. The wall-associated receptor kinase 5-like gene (AtWAKL5) is also involved in the response to pathogenic infection in *A. thaliana* [43]. One reason for less lignin deposition and peroxidase activities [33, 44] is the laccase (LCC) gene family [15, 45]. Therefore, the high expression of LCC genes may be associated with susceptibility to *G. boninense*. An earlier report identified at least 17 LCC genes in *A. thaliana* genomes [46]. However, no information is available about the association of LCC gene expression and the oil palm response against *G. boninense*. Additionally, it is necessary to explore the other genes involved in root growth, plant development, and biotic stress response to BSR disease in oil palm.

Evaluating the physiological, biochemical, and molecular processes associated with resistance responses against *G. boninense* may benefit oil palm breeding programs and may develop *G. boninense*-resistant oil palm varieties. Therefore, evaluating these factors during the internal symptoms of BSR disease in the seedling stage of oil palm is necessary. Hence, this study aims to (1) assess the effects of *G. boninense* infection on seedling growth and development, (2) group seedling responses based on the *Ganoderma* disease index (GDI) into either susceptible or resistant seedlings, and (3) evaluate the changes in root lignin and SA content, peroxidase activities, and relative expression of genes in the roots of oil palm seedlings after early symptoms of *G. boninense* infection.

## Results

### Symptom occurrence and disease incidence (DI)

The inoculated seedlings showed infection symptoms ranging from 5.6–6.0 months after artificial inoculation (MAI). Progeny populations derived from crosses between Dumpy *dura* 27 × AVROS *pisifera* and Dumpy *dura* 8 × AVROS *pisifera* (C01 and C03) showed the earliest symptom occurrence (5.6 and 5.8 MAP, respectively, Table 1). In addition, other seedling populations (C02, C05, C07, and C08) showed initial symptoms at 6.0 MAI (Table 1). DIs among the inoculated seedlings ranged from 16.9%–42.5%. The progeny populations derived from crosses between Deli *dura* and Yangambi *pisifera* (C08) had the lowest DIs (16.9%, Table 1). Moreover, the C01 and C05 seedling populations had the highest DIs (35.6 and 42.5, respectively, Table 1). The DIs of the other seedling populations (C02, C03, and C07) were between those of C08 and C05 (Table 1). There was no correlation between the time of symptom occurrence and the percentage of disease incidence.

**Table 1:** Genetic background of utilized plant materials and resistance responses after *Ganoderma boninense* inoculation to oil palm seedlings

**Accession code**	**Genetic background of progeny**	**Symptom Occurrence (month)**	**Disease Incidence (%)**	***Ganoderma*** **Disease Index**	**Resistance Response**
C01	Dumpy *dura* 27 x AVROS *pisifera*	5.6 a	35.6 b	116.0 b	Susceptible
C02	Dumpy *dura* 50 x AVROS *pisifera*	6.0 a	32.7 c	106.6 c	Susceptible
C03	Dumpy *dura* 8 x AVROS *pisifera*	5.8 a	27.7 e	90.3 e	Resistant
C05	Dumpy *dura* 29 x AVROS *pisifera*	6.0 a	42.5 a	138.5 a	Susceptible
C07	Deli *dura* x La Mé *pisifera*	6.0 a	28.7 d	96.5 d	Resistant
C08	Deli *dura *x Yangambi *pisifera*	6.0 a	16.9 f	56.8 f	Resistant
Average		5.9	30.7	100	

The mean values were taken from 5 replicates with 7–10 seedlings for each replicate. The total number of seedlings showing symptoms was 277, with an average of 52.83 seedlings per accession. Mean values of the recorded responses followed by different letters were significantly different for each recorded characteristic based on Tukey's Studentized Range (HSD) test at *p* < 0.05

### Ganoderma disease index (GDI) and resistance response

Table 1 also shows that the GDI for the inoculated seedlings ranged from 56.8–138.6. Based on their GDI value, the C01, C02, and C05 seedling progenies were placed into the *G. boninense* susceptible group, with GDI values ranging from 106.6–138.5 (Table 1). C05 seedling progeny were the most susceptible to *G. boninense* infection, with a GDI value of 138.5 (Table 1). The other three progenies (C03, C07, and C08) were grouped as resistant to *G. boninense*, with GDI values ranging from 56.8–90.3 (Table 1). The C08 seedling progeny had the most resistance to *G. boninense,* with a GDI value of 56.8 (Table 1). Therefore, the progeny between Dumpy *dura* 27 × AVROS *pisifera* (C01), Dumpy *dura* 50 × AVROS *pisifera* (C02), and Dumpy *dura* 29 × AVROS *pisifera* (C05) yielded susceptible progenies to *G. boninense* infection (Table 1). The seedlings derived from crosses between Dumpy *dura* 8 × AVROS *pisifera* (C03), Deli *dura* x La Mé *pisifera* (C07), and Deli *dura* x Yangambi *pisifera* (C08) yielded resistant progenies (Table 1).

### Ganoderma boninense infection symptoms in oil palm

**Fig. 1:**
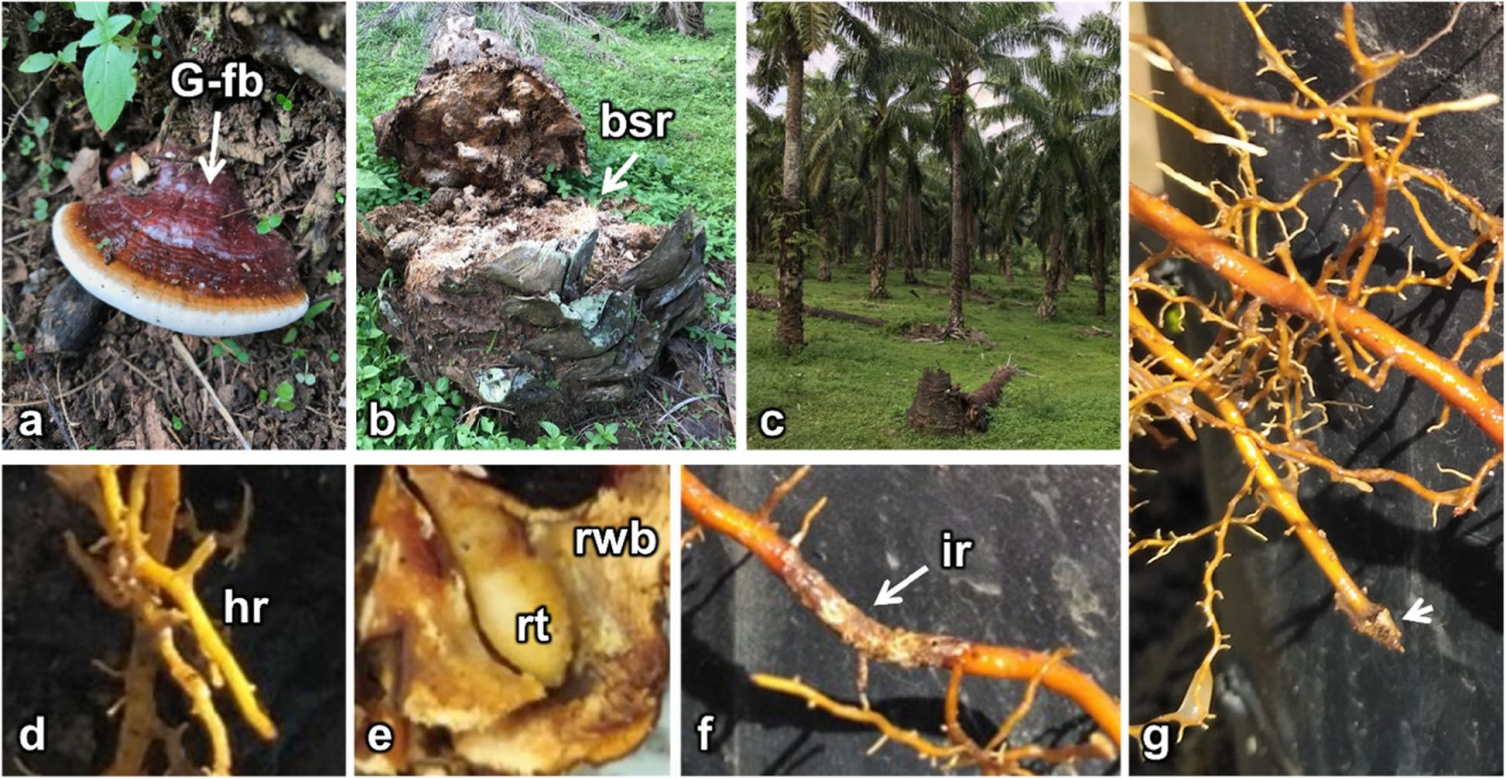
Examples of basal stem rot (BSR) symptoms observed in the field and nursery. **a** Basal stem rot disease infected oil palm from *Ganoderma boninense*, showing *Ganoderma* fruiting bodies (G-fb) growing at the basal stem. **b** The severe and late-stage symptoms of BSR disease infection caused the stem to die, and the tree to fall. **c** The field condition of oil palms with BSR infection. **d** Healthy root (hr), (**e**) a root tip (rt) penetrating rubber woodblock (rwb) used for inoculating oil palm seedlings with *G. boninense.* **f** Oil palm root infected with *G. boninense*. **g** The arrest of root tip growth in an infected root of a susceptible oil palm seedling

A sign of *G. boninense* infection in adult oil palm trees under field conditions includes the presence of a *Ganoderma* fruiting body at the base of the trunk (Fig. 1a). The BSR disease occurs under severe infestation and eventually, the oil palm tree dies (Fig. 1b). Most oil palm provenances in the same plantation area may become infected with BSR disease (Fig. 1c). In this experiment, most inoculated seedlings scored 1 for internal infection symptoms based on the standard scoring for *G. boninense* disease development. There was no *Ganoderma* fruiting body growing on the base of oil palm seedlings until the end of the experiments (7 months after initial infection). A visible symptom of *G. boninense* infection in the susceptible seedlings appeared as brown necrosis in the bifid leaf of the inoculated oil palm seedlings.

*G. boninense* inoculation of resistant seedlings did not affect root apical meristem growth and development. The roots of the tested resistant seedlings thrived up to seven months. Figure 1d shows an example of healthy root development of the resistant oil palm seedlings, and Fig. 1e shows a root tip of the resistant oil palm seedling penetrating a rubber woodblock (RWB) source of *G. boninense* inoculation. Contrary to the resistant seedlings, *G. boninense* inoculation of susceptible seedlings affects root apical meristem growth and development. Early infection symptoms in the susceptible oil palm seedlings roots include white mycelia, necrotic and damaged regions in the infected roots, and apical meristem root growth arrest. The root tip did not undergo elongation or cell division in severe infections. Figure 1f shows an example of *G. boninense-*infected roots of a susceptible oil palm seedling, and Fig. 1g shows the arrest of tip growth of an infected roots.

### Growth and biomass yield of G. boninense-infected oil palm seedlings

**Fig. 2:**
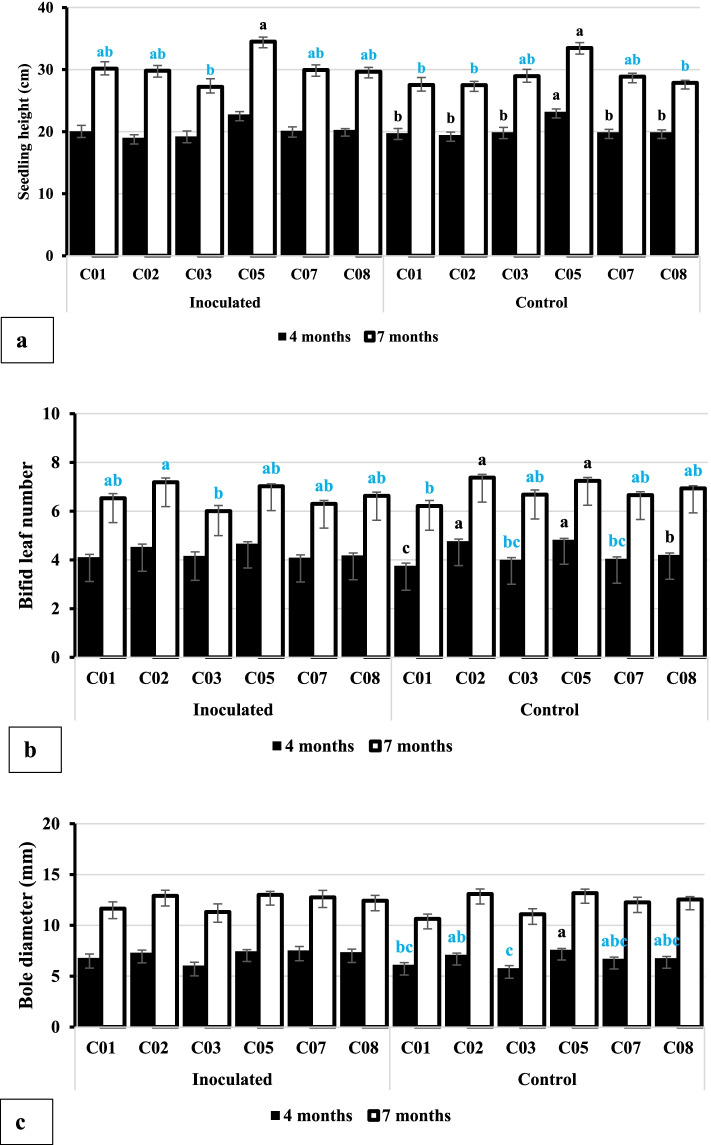
Effects of *Ganoderma boninense* infection on aerial growth of oil palm seedlings. **a** Seedling height, (**b**) bifid leaf number, and (**c**) bole diameter of oil palm seedlings. Note: For each observation parameter, the average of 4 months and 7 months after planting with the same lowercase letter shows that they were not significantly different based on a t test at α = 0.05. Similarly, the average value of each accession with the same lowercase letter shows that they were not significantly different based on the Tukeys's Honest Significant Difference (HSD) test at α = 0.05. The t test was conducted using StatPlus, while ANOVA and HSD used Statistical Tool for Agricultural Research (STAR) program

**Fig. 3:**
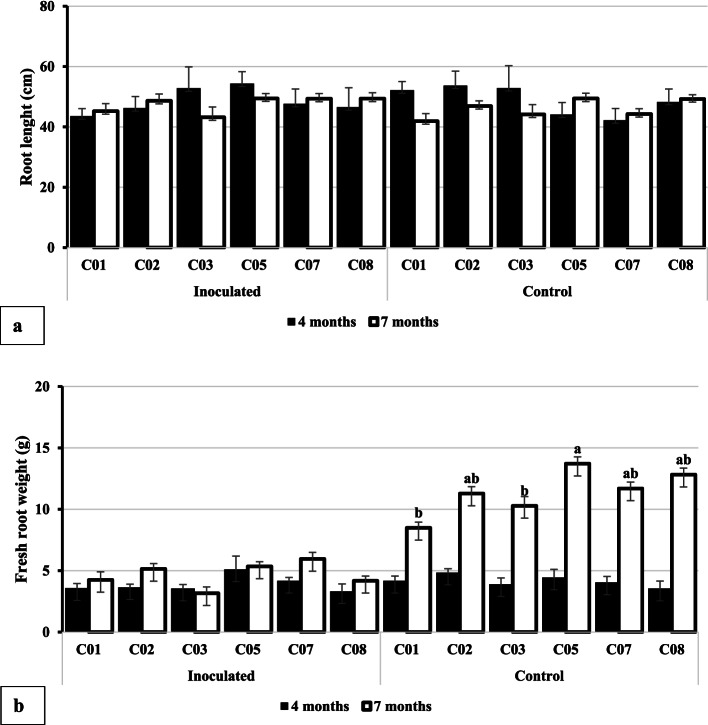
Effects of *Ganoderma boninense* infection on root growth and biomass of oil palm seedlings. **a** Root length and (**b**) fresh root weight. Note: For each observation parameter, the average of 4 months and 7 months after planting with the same lowercase letter indicates that they were not significantly different based on a t test at α = 0.05. Similarly, the average value of each accession with the same lowercase letter shows that they were not significantly different based on the Tukeys's Honest Significant Difference (HSD) test at α = 0.05. The t test was conducted using StatPlus, while ANOVA and HSD used Statistical Tool for Agricultural Research (STAR) program

The statistical analysis results of the statistical analysis indicated that only the main effects of genotype, inoculation, or observation times were statistically significant. Therefore, only the main effects of genotype, inoculation, and observation times are presented in Figs. 2 and 3. Figure 2 shows the effects of the *G. boninense* infection effects on the growth and development of the aerial seedlings of oil palm at four and seven months after planting (MAP). Figure 3 presents the root growth and biomass at four and seven MAP.

At both four and seven MAP, the average seedlings height (SH), bifid leaf number (BLN), and bole diameters (BD) of oil palm seedlings from either the control or infected plot were presented in Fig. 2. However, the average SH of the C05 seedlings was the highest, while that of the other seedlings was almost the same (Fig. 2a). *Ganoderma* infection did not affect the SH of C05 seedlings accessions. On the contrary, the BLN at four MAP was not significantly different among seedling populations, while at seven months MAP. C01 had the lowest SH (Fig. 2b). There was a potential decrease in BLN at 7 MAP in all accessions, except C01. The average BD of C03 seedlings in control plot was bigger than the others (Fig. 2c), but no significant difference were observed among accessions in control and inoculated seedlings four and seven MAP (Fig. 2c).

The average root length (RL) of oil palm seedlings in the control was not significantly different from that of infected plots (Fig. 3a) at four and seven MAP. However, the RL of the C01 and C03 seedlings were the shortest, and those of the C08 seedlings were the most extended seven MAP (Fig. 3a). The average fresh root weight (FRW) of oil palm seedlings in control was significantly different from the infected plot (Fig. 3b). Similarly, the average FRW of oil palm seedlings in the control plot at four MAP was not significantly different from that in the infected plot (Fig. 3b). Meanwhile, a higher average FRW was presented in the inoculated plot at seven MAP (Fig. 3b). The average FRW of the C05 seedlings was the highest, and that of the C01 seedlings was the lowest at four MAP (Fig. 3b).

### Lignin content, peroxidase activity, and salicylic acid accumulation

**Fig. 4:**
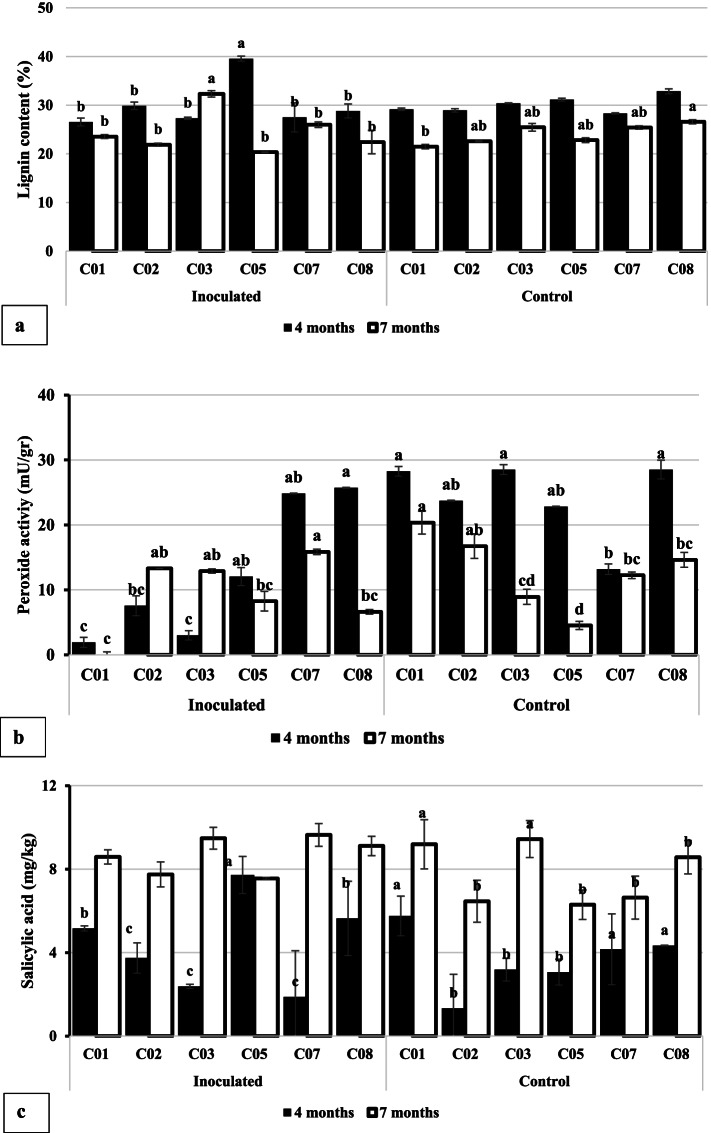
Effects of *Ganoderma boninense* infection on physiological responses of oil palm. Lignin content (**a**), peroxidase activity (**b**), and salicylic acid content (**c**) of oil palm seedlings at four months and seven months after planting (MAP). Note: For each observation parameter, the average of 4 months and 7 months after planting with the same lowercase letter shows that they were not significantly different based on a t test at α = 0.05. Similarly, the average value of each accession with the same lowercase letter shows that they were not significantly different based on the Tukeys's Honest Significant Difference (HSD) test at α = 0.05. The t test was conducted using StatPlus, while ANOVA and HSD used Statistical Tool for Agricultural Research (STAR) program

At four months and seven months after planting, the average root lignin content (LIGNIN) of oil palm seedlings from the control plot differed significantly from the inoculated plot for all accession (Fig. 4). However, individual accessions were significant at four months and seven MAP in the inoculated plot. At four months, the control plot did not exhibit a significant difference (Fig. 4a). The average root lignin content of the C05 seedlings inoculated with *Ganoderma* was the highest average root LIGNIN four MAP, but the lowest LIGNIN seven MAP (Table 4a). Upon infection of *G. boninense*, the susceptible seedlings (C05) significantly decrease their LIGNIN seven MAP. On the contrary, upon infection with the fungi, resistant seedlings (C03) significantly increase LIGNIN seven MAP (Fig. 4a).

At both four months and seven MAP, in general, the average root PRX activity of oil palm seedling samples from the control plot was higher than that from the infected plot (Fig. 4b). In the inoculated plot, the average root PRX activity of the C08 seedlings was the highest, and that of the C01 seedlings was the lowest at four MAP (Fig. 4b). The C07 and C08 shows remained high activities at 4 MAP in the inoculated plot. However, the average PRX activity of the C08 seedlings was the lowest, and that of the C07 seedlings was the highest at seven MAP in the inoculated plot (Fig. 4b). At seven MAP, all accessions had lower PRX activity, except for C02 and C03, which had higher PRX.

At four months and seven MAP, there was an increased average root SA content of oil palm seedlings in control plots and infected plot (Fig. 4c). A higher average root SA content was presented at seven MAP than at four months (Fig. 4c). In the inoculated plot, the average root SA content of the C05 seedlings was the highest, and that of the C07 seedlings was the lowest at four MAI (Fig. 4c). Otherwise, the average root SA content of the C05 seedlings was the highest, and that of the C07 seedlings was the lowest at seven MAI. The C01, C07, and C08 accessions have high SA while C02, C05, and C05 have low SA accumulation at four MAP in the control plot. The average roots SA content of the C01 and C03 were higher at seven MAP while other C02, C05, C07, and C08 were found lower in the control plot seedlings.

### Expression of genes involved in oil palm root seedlings after *Ganoderma boninense* infection

**Fig. 5:**
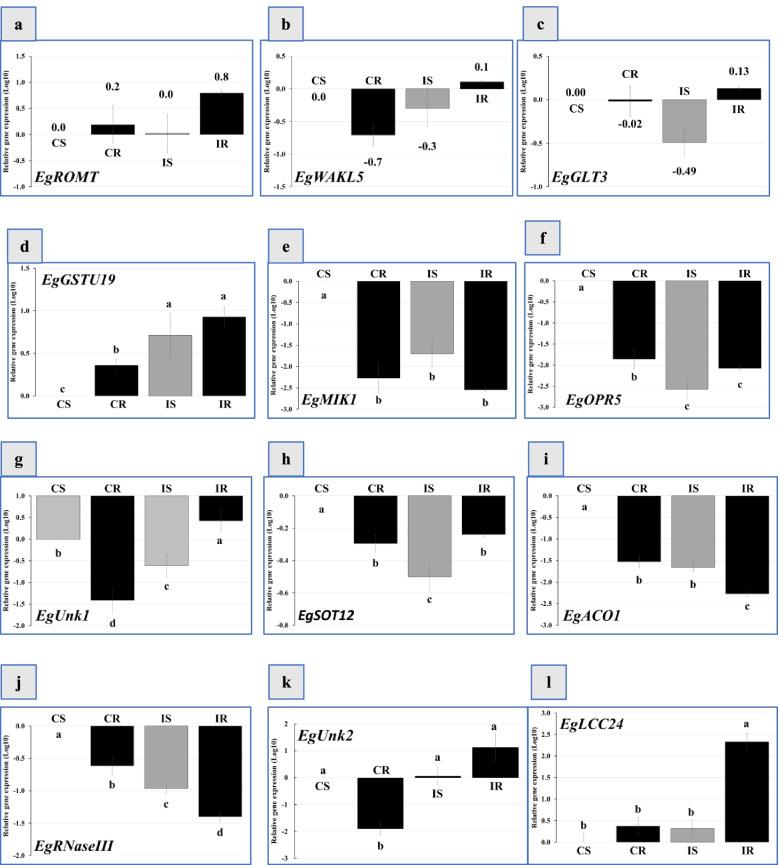
Relative normalization of 12 genes associated in *Ganoderma boninense* infection in oil palm roots. The relative gene expression was measured in the roots of oil palm seedling at the time of symptoms occurrences. The expression patterns of targeted genes were normalized by the Actin-101 gene expression level. qPCR was carried out on susceptible control (SC), control resistant (CR), inoculated susceptible (IS), and inoculated resistant root samples. The statistical analysis was carried out by comparing the level of normalized relative expression using Log10 fold change at each different treatment to control samples using the F test analysis to determine the level of homogeneity of variance, followed by the t test to determine the significance. The levels of expression that differed significantly compared with controls were measured according to *p* < 0.05. The difference of expression level for each gene were analyzed using Least Significant Difference (LSD) test at α = 0.05 was presented in STAR program

Because of resources and the infected oil palm root tissues limitation, relative expression analysis of 12 genes were carried out for C01 (susceptible-) and C03, C07, and C08 accessions (resistant). Results of the qPCR analysis showed that the relative expressions of EgROMT, EgWAKL5, and EgGLT3 genes were not significantly different among the evaluated samples (Fig. 5a, b, c). Relative gene expression of EgGSTU19 was significantly up-regulated in the uninoculated resistance seedlings. In contrast, the relative gene expression of EgGSTU19 in the inoculated resistant samples was not statistically different from the uninoculated control (Fig. 5d). Relative gene expression of EgMIK1 and EgOPR5 was significantly down-regulated in uninoculated resistance seedlings, while the relative gene expression of both genes in the inoculated resistant samples was not statistically different to the uninoculated control (Fig. 5e, f).

Relative gene expression of the EgUnk1 (an unidentified gene 1) and EgSOT12 were significantly increased in the control uninoculated resistance than the susceptible samples (Fig. 5g, h). However, relative gene expression of the EgUnk1 and EgSOT12 were significantly reduced in the inoculated resistance than the susceptible samples (Fig. 5g, h).

The EgACO1 and EgRNaseIII relative gene expression significantly increased in the control uninoculated and inoculated resistance than the susceptible samples (Fig. 5i, j). The EgUnk2 relative gene expression was significantly down-regulated in inoculated resistant samples, while the relative gene expression among inoculated control of both the susceptible and resistant samples were not significantly different (Fig. 5k). The EgLCC24 relative gene expression was significantly higher in inoculated resistant than the control uninoculated susceptible samples, while the relative gene expression among uninoculated control of both the susceptible and resistant samples were not significantly different (Fig. 5l).

There are a significant relationship of the evaluated genes based on Pearson's coefficient correlation (Table 2). Most of the genes being assessed were positively correlated, except for the EgLCC24 gene which was negatively correlated with EgACO1. (Table 2). We found a positive correlation between EgMIK1 with EgRNaseIII; EgWAKL5 with EgSOT12; EgRNaseIII, EgROMT, EgGLT3, with EgOPR5; and GLT3 with EgSOT12, EgROMT, EgWAKL5, EgGSTU19, EgOPR5, and EgLCC24; EgLCC24 with EgSOT12 and EgUnk1; and EgUnk1 with EgUnk2 (Table 2).

**Table 2:** Pearson’s correlation analysis among relative expressions levels of evaluated genes associated with *Ganoderma boninense* infection in oil palm root seedlings

Genes	EgMIK1	EgUnk1	EgUnk2	EgACO1	EgRNaseIII	EgROMT	EgGLT3	EgWAKL5	EgGSTU19	EgOPR5	EgLCC24
EgSOT12	0.42	0.60 *	0.14	- 0.35	0.29	0.5	0.83 **	0.72 **	0.34	0.42	0.73 **
EgMIK1		0.04	0.14	0.38	0.81 **	- 0.14	0.01	0.53	- 0.40	0.10	- 0.03
EgUnk1			0.60 *	- 0.45	- 0.16	0.18	0.45	0.09	0.09	0.24	0.71 **
EgUnk2				0.06	0.01	- 0.05	0.05	0.09	- 0.40	0.25	0.09
EgACO1					0.27	- 0.21	- 0.43	0.07	- 0.37	0.24	- 0.79 **
EgRNaseIII						0.07	0.07	0.60 *	- 0.31	0.04	- 0.16
EgROMT							0.86 **	0.67 *	0.50	0.78 **	0.23
EgGLT3								0.72 *	0.62 *	0.61 *	0.63 *
EgWAKL5									0.09	0.65 *	0.14
EgGSTU19										0.09	0.52
EgOPR5											- 0.05

The * or ** shows a significant (*p* ≤ 0.05) or a highly significant (*p* ≤ 0.01) of the Pearson’s correlation coefficient, respectively

### Correlation among lignin, peroxidase activity, salicylic acid content, growth, and biomass yield

Table 3 shows the results of the Pearson correlation analysis among seedling growth and development and seedling responses against *G. boninense* infection before the seedlings showed any infection symptoms (4 MAP). Table 3 shows that the GDI had no significant correlation between seedling growth and seedling responses. The root lignin content had a significant correlation with SH, BLN, and FRW. However, the lignin content did not have a significantly correlate with BD, RL, SA, or PRX (Table 3). The PRX and SA did not correlate with components of seedling growth and development or seedling responses (Table 3) before the inoculated seedlings showed internal infection symptoms (4 months after planting).

**Table 3:** Pearson correlation coefficient among defences response in the inoculated oil palm seedlings four months after planting

Seedling responses	Seedling growth and development					Seedling responses			
	BLN	BD	RL	FRW	GDI		LIGNIN	PRX	SA
SH	0.52	0.45	0.5	0.85 *	0.52		0.86 *	0.23	0.78
BLN		0.38	0.46	0.62	0.58		0.87 *	-0.16	0.60
BD			-0.2	0.44	0.10		0.42	0.69	0.35
RL				0.62	0.31		0.65	-0.07	0.18
FRW					0.75		0.85 *	0.10	0.45
GDI							0.60	-0.51	0.37
LIGNIN								0.05	0.74
PRX									-0.02

*SH* seedling height, *BLN* bifid leaf number, *BD* bole diameter, *RL* root length, *FRW* fresh root weight, *GDI Ganoderma* disease index, *LIGNIN* root lignin content, *PRX* root peroxidase activity, and *SA* root salicylic acid content. The * or ** shows a significant (*p* ≤ 0.05) or a highly significant (*p* ≤ 0.01) of the Pearson’s correlation coefficient, respectively

**Fig. 6:**
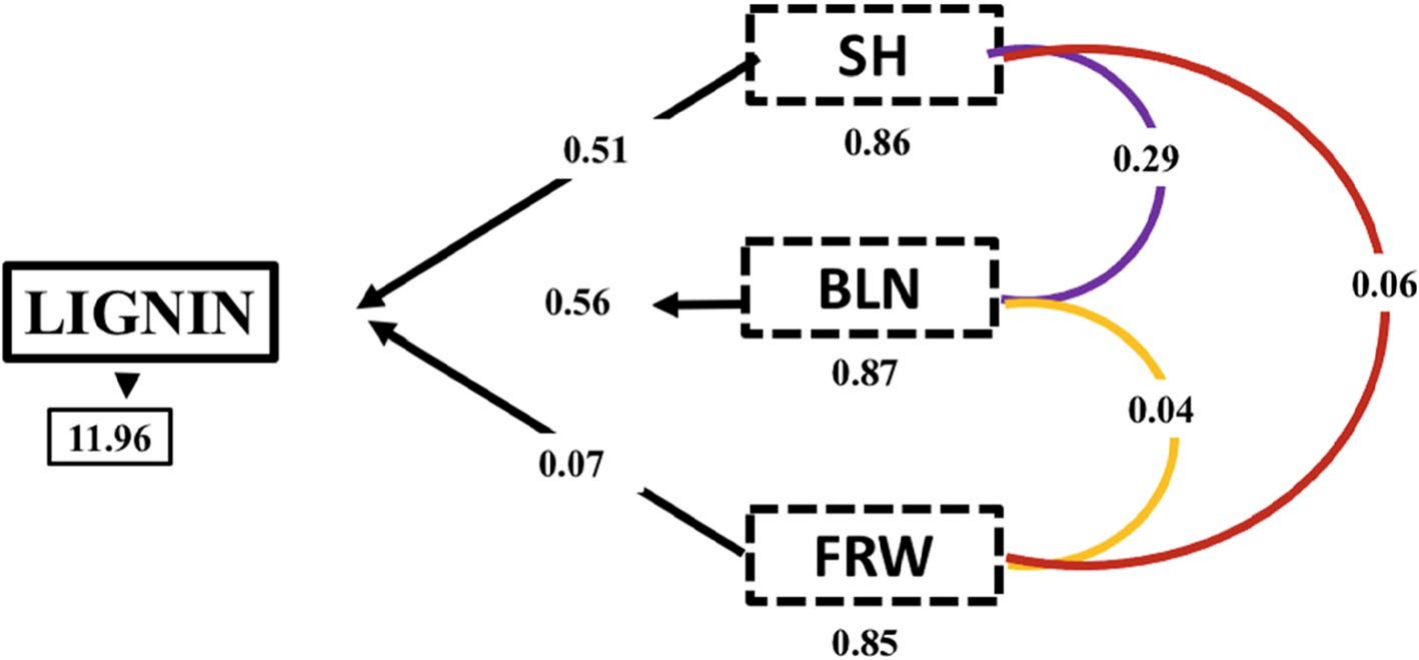
Direct and indirect effect of growth and development oil palm seedlings toward lignin content. The characters of seedlings height (SH), bifid leaf number (BLN), and fresh root weight (FRW) toward lignin content (LIGNIN) at four months after planting with infected *Ganoderma boninense*. Path analysis conducted using R 4.0.5 version

Based on path analysis, there is a significant indirect effect on SH (0.51), BLN (0.56), and FRW (0.07) towards LIGNIN with a correlation value of 0.86, 0.87. and 0.85 at *p*-value of 0.05, respectively (Fig. 6). The SH, BLN, and FRW were not affected by *Ganoderma* infection aged 4 months after planting (Figs. 2 and 3), but these three characters directly affected lignin content (Fig. 6). There was no direct effect of the growth and development of oil seedlings on lignin at seven months after planting [data not presented].

Table 4 shows the results of the Pearson correlation analysis among seedling growth and development and seedling responses against *G. boninense* infection after the seedlings showed visible infection symptoms (7 months after planting). GDI did not have a correlation with the growth and response of *Ganoderma* resistance to oil palm seedlings (Tabel 4). Table 4 shows that the FRW had a significant correlation with SH, BD, and RL. BLN had a negative correlation with SA content and LIGNIN, while PRX had no significant correlation with either seedling growth and development of seedling responses (Table 4). On the other hand, root lignin content negatively correlated with BLN and FRW (Table 4).

**Table 4:** Pearson correlation coefficient among defense response in the inoculated seedlings seven months after planting

Characters	BLN	BD	RL	FRW	GDI	PRX	LIGNIN	SA
SH	0.65	0.67	0.61	0.94 **	0.67	-0.43	-0.77	-0.71
BLN		0.74	0.63	0.68	0.40	-0.34	-0.88 *	-0.91 *
BD			0.94 **	0.82 *	0.24	-0.10	-0.74	-0.51
RL				0.83 *	0.00	-0.27	-0.76	-0.33
FRW					0.42	-0.45	-0.86 *	-0.58
GDI						0.15	-0.31	-0.67
PRX							0.50	0.35
LIGNIN								0.74

*SH* seedling height, *BLN* bifid leaf number, *BD* bole diameter, *RL* root length, *FRW* fresh root weight, *GDI Ganoderma* disease index, *LIGNIN* root lignin content, *PRX* root peroxidase activity, and *SA* root salicylic acid content. The * or ** shows a significant (*p* ≤ 0.05) or a highly significant (*p* ≤ 0.01) of the Pearson’s correlation coefficient, respectively

## Discussion

### Occurrence and internal symptoms of *G. boninense *infection in oil palm seedlings

The ability to evaluate responses against *G. boninense* at the seedling level is essential in the development of new BSR disease resistance in oil palm varieties. Unfortunately, the aerial symptoms of *G. boninense* in oil palm seedlings were not clear. Therefore, it will be challenging to determine the response differences among oil palm seedling populations based on the aerial symptoms of *G. boninense* infection. Several factors have affected the success of oil palm seedling artificial inoculation with *G. boninense*, such as the virulence of *G. boninense* isolates, preinfected RWB, and microclimates of nursery factors [47].

Symptoms of *G. boninense* infection are more observable in the roots than in the aerial parts of oil palm seedlings. However, observation of root infection requires destructive observation of the sampled seedlings. Breton et al. [48] reported that under optimized inoculation conditions, they observed *G. boninense* infection in the roots of oil palm seedlings three months after the inoculation of germinated seeds. There was a sign of root apical meristem growth arrest because of *G. boninense* infection in susceptible oil palm roots. On the other hand, the apical meristem of the root of resistant oil palm seedlings grew well, and they penetrated the *G. boninense*-infected RWB without any damage.

The early infection of *G. boninense* in roots of inoculated oil palm seedlings occurs through root contact with inoculum sources [49]. The biotrophic phase of the *G. boninense* interaction is characterized by white hyphae on the infected oil palm roots. The necrotrophic phase of infection is indicated by the production of cell wall-degrading enzymes (CWDEs) by *G. boninense* that destroys the oil palm roots [50]. The three oxidative enzymes of *G. boninense*, *manganese peroxidase (MnP), laccase (LCC),* and *lignin peroxidase (LiP),* are ligninolytic enzymes of CWDEs [28]. Oil palm lignin consists of a high percentage of syringyl (S) units to guaiacyl (G) ratio (S/G) at either four weeks post-inoculation (wpi) or eight wpi [34]. On the other hand, wood lignin contains more G units resistant to *G. boninense* infection than S units [51]. Investigations to evaluate the ratios between the S and G types of lignin in the roots of oil palm accessions and their association with resistance responses against *G. boninense* might be essential in the future.

### Ganoderma disease index and responses to G. boninense infection in oil palm seedlings

The average value of DIs for the tested population was used to determine the resistance responses against *G. boninense* infection by determining the GDI. Hence, the GDI facilitates the grouping of oil palm progenies that are heterozygous and heterogeneous [52] into either resistant or susceptible groups [1]. Based on their GDI value, three out of the six tested seedlings were placed into the group that was susceptible to *G. boninense* (C01, C02, and C05), and the other three were placed into the resistant group (C03, C07, and C08). Therefore, the progeny crosses between Dumpy dura 27 × AVROS *pisifera* (C01), Dumpy dura 50 × AVROS *pisifera* (C02), and Dumpy dura 29 × AVROS *pisifera* (C05) yielded seedlings susceptible to *G. boninense* infection. Dumpy dura 29 × AVROS *pisifera* (C05) seedlings were the most susceptible to *G. boninense* infection. The progenies between Dumpy dura 8 × AVROS *pisifera* (C03), Deli *dura* x La Mé *pisifera* (C07), and Deli dura x Yangambi pisifera (C08) were resistant to *G. boninense* infection. However, the Deli *dura* x Yangambi *pisifera* (C08) cross were the most resistant.

The progenitors of oil palm developed in Indonesia and Southeast Asia mostly date back to crosses of the *dura* type of oil palm introduced to Bogor Botanical Garden in 1848 and the *pisifera* type of oil palm from Africa [53]. Subsequently, the *dura* type of oil palm derived from the Bogor Botanical Garden [53] was known as Deli *dura* [54, 55]. Oil palm genetic materials known as the Yangambi and La Mé populations are more resistant to *G. boninense* than those of Dura Deli [4, 56]. Little is known about why Deli Dura-derived oil palm varieties are susceptible to *G. boninense.* Possible reasons include the narrow genetic background or the extensive homozygosity of the Dura Deli-derived oil palm varieties [57]. In this study, the genetic background of C01, C02, C03, and C05 accessions were the same but resulted in different levels of resistance to *G. boninense*. It showed variability of resistance at the same genetic background. There is also the variability of resistance among progenies derived from the same or different Deli *dura* sub populations. All the dura parents of accessions used in this study were Deli *dura*, in which the *dura* parents of C01, C02, C03, and C05 were derived from the same Deli *dura* sub populations of Dumpy, while the C07 and C08 were derived from other Deli *dura* sub populations. The findings supported a report that stated that a few crosses among Deli *dura* were also relatively resistant against *G. boninense* infection [13].

Hybridization between certain *dura* × *pisifera* was reported to increase the relative resistance against *G. boninense* infection of the progenies compared with the *dura* self progenies [13]. These data indicated that extensive homozygosity might be associated with susceptibility against *G. boninense* infection. Based on the previous results of genetic data analysis, the Deli *dura*-derived oil palm varieties are genetically distantly related to the Yangambi oil palm varieties [55, 57]. Further studies in this direction should shed light on the mechanisms of either resistance or susceptibility of oil palm against *G. boninense* infection. Establishing the genetic control of resistance against *G. boninense* infection in oil palm progenies requires the availability of procedures for evaluating the resistance response against *G. boninense* infection at the seedling level and using artificial inoculation [48]. The methods described in this study may be used to identify *G. boninense* isolates with virulence, as suggested by Breton et al. [48], and to determine the resistance responses of oil palm accessions at the seedling level.

### Growth, development, and root biomass yield in infected oil palm seedlings

In both infected and control plots, the aerial growth of the oil palm seedlings at four months after planting was homogeneous. At seven months after planting, the average height of the C05 seedlings was the highest, while that of the other seedlings was almost the same height. The BLN of the C01 seedlings seven months after planting was the lowest. Moreover, for the BD of oil palm seedlings at seven months after planting, C02 and C05 had the largest bole diameters, and the C01 seedlings had the smallest bole diameters. All these data indicated that *G. boninense* infection in oil palm barely affected the aerial growth of the evaluated seedlings. Compared with the resistance response at C05 and C03 (Table 1), there was no strong association between SH, BLN, and BD against *Ganoderma boninense* infection in both of resistant and susceptible seedlings.

No differential responses in RL were recorded between the control and infected plots at four and seven months after planting. Although the average FRW of oil palm seedlings in the control was similar to that in the infected plot at four months after planting, a higher average FRW was seen in the control plot than in the inoculated plot at seven months, indicating that *G. boninense* infection inhibited the root growth and development of oil palm seedlings. The interesting fact about growth, development, and root biomass yield in infected oil palm seedlings is that there is a potential for decreasing LR and FRW root biomass in inoculated seedlings. This decrease did not occur in susceptible seedlings only, but also in *Ganoderma*-resistant seedlings. C05 seedlings had the highest growth, development, and root biomass yield, but had the lowest susceptibility to *Ganoderma*. Meanwhile, the performance of C03-resistant seedlings such as growth, development, and root mass were relatively low. It seems difficult to find out the effect of *Ganoderma* infection per accession. In general, *G. boninense* infection has the potential to inhibit the growth rate, development, and biomass of oil palm roots which is in the below of the soil surface.

Differences in root biomass of RL and FRW in seedlings infected with *G. boninense* became the first important indicator to predict the initial defense response to BSR disease infection in oil palm. Leaf vegetation, SH, and BD of the oil palm are the second indicator that need to be considered for growth. It is indicated an initial defense response against the presence of *G. boninense* infection.

In this study, oil palm seedling growth and development upon inoculation with *G. boninense* were similar to previous studies results [32]. The aerial parts of the inoculated seedlings were generally unaffected by *G. boninense* inoculation. However, the changes in root biomass of the inoculated seedlings illustrated the effect of *G. boninense* infection in early oil palm development [34]. Since *G. boninense* is a hemi biotrophic fungus, understanding the transition processes between the biotrophic phase and necrotrophic phase is essential for the success of *G. boninense* and oil palm seedling interactions [49]. It has been reported that the effector proteins, host defense mechanisms, and infection processes are different among biotrophic, necrotrophic or hemi biotrophic pathogens [58, 59].

### Physiological responses of oil palm seedlings to G. boninense infection

*Ganoderma boninense* is a hemi biotrophic fungus [60]. Therefore, the interaction between *G. boninense* and oil palm roots occurs in two phases: the biotrophic phase and the necrotrophic phase. According to Bahari et al. [19], the biotrophic phase occurs as early as 3–7 days after root infection, and it is indicated by the increased expression of many defense-related genes (PR-protein, protease inhibitor, PRR protein, chitinase, and expansin). The transition to the necrotrophic phase can occur as early as eleven days after infection [19]. The infected oil roots start to activate necrotrophic defense mechanisms. However, the necrotrophic defense eventually defers at a later stage to the challenges of *G. boninense* in susceptible oil palm seedlings [19].

Our studies were conducted during the 4–7 months period after inoculation. Based on Bahari et al. [19], they should have already entered the necrotrophic stage. In this study, the root lignin contents of the inoculated and uninoculated control plots were different. Differences in lignin content became the third important aspect finding in this study, after root biomass (RL and FRW) and vegetative leaf and bole of oil palm seedlings. The lignin content is thought to be related to the existence of the physical defense system in the early stages of BSR disease infection.

Lignin content is important in the early defense against *G. boninense* infection [61]. At a later stage of infection, cell wall lignification was not sufficient in protecting the oil palm root from *G. boninense* infection [19, 34]. Moreover, the root PRX activities showed same response patterns than the root lignin content. At both four and seven months after planting, *G. boninense* infection reduced the average root PRX activity of oil palm. Peroxidase (PRX) is one of the enzymes involved in lignin biosynthetic processes [38, 61]. Although it successfully inhibits fungal growth, the high PRX activity is not sufficient in preventing infection when *G. boninense* switches to the necrotrophic phase [61].

*Ganoderma boninense* infection did affect root SA content at either four months or seven months after planting. Each seedling population (control and *G. boninense-*infected) had a different level of root SA content at either four or seven months after planting. However, the root SA content in oil palm seedlings was associated with the age of seedlings, and it was not because of the *G. boninense* infection since seedlings at seven months after planting (control and *G. boninense-*infected) had higher SA content than those at four months.

At the beginning of BSR disease infection, oil palm seedlings in susceptible seedlings (C01, C02, and C05) produced higher root SA than resistant seedlings (C03, C07, and C08), but at the end of the observation after symptoms appeared, root SA production in seedlings resistance increased significantly the production of root SA. The accumulation of root SA in susceptible seedlings tends to be faster or responsive to the presence of BSR disease infection, especially in C05. However, in C03 and C07 resistant seedlings there was a very high increase in SA accumulation after symptoms appeared. It seems that C05 had the best performance in the physical growth and development of seedlings, but physiologically and biochemical responses (LIGNIN, PRX, and SA) decreased their biochemical defense activity. On the other hand, the physical defense against infection with BSR disease in C03 was relatively decreased, but the physiological and biochemical defense was very responsive to *G. boninense*. There is a mutually supportive role among physical, physiological, and biochemical defense in responding to the *Ganoderma* infection.

A previous report stated that SA defense pathway activation is indicated by the induction of various defense-related genes [62]. Moreover, pathogen-infected plants induce a wide variety of physiological and biochemical responses [63]. One of the plant biochemical defense responses to diseases is the increase in SA accumulation [63, 64], which functions as a signal transduction compound. In this study, the high accumulation of root SA content was not always followed by an increase in peroxidase activity, which indicated that disease resistance mechanisms are very diverse among plant species and genotypes. The findings of this study indicated that the *G. boninense* resistance mechanisms in some of the seedling populations might be an independent mechanism of the SA defense pathway.

### Targeted gene expression in infected oil palm roots

Oil palm has genes involved in biotic stress, but its physiological function in the resistance response to *Ganoderma* still requires further information. In addition, some of the genes involved in the infection of these pathogens have been studied in more depth for their relative gene expression and physiological functions in susceptible and resistant *Ganoderma* seedlings. Our findings indicated four gene groups based on gene response and expression in the interactions of *G. boninense* and oil palm seedlings. They were classified into (1) highest expressed genes in the resistant seedlings, (2) highest expressed genes in the susceptible seedlings, (3) not significantly different genes to *Ganoderma* infection, and (4) candidate genes for molecular biomarkers of *Ganoderma* infection.

First, the highest expressed genes, EgLCC24, were in the inoculated resistant seedlings. EgLCC24 gene expression was highly expressed in Ganoderma-infected seedlings, especially resistant seedlings indicated that the gene activated by *Ganoderma* infection. Plant LCCs are more highly glycosylated than fungal laccases [46]. *G. boninense* also has LCC genes in its genome [4, 65]. The function of LCC genes from *G. boninense* is probably associated with lignin degradation in the infected root tissues of oil palm [60, 66]. In different Ganoderma species (*G. tsugae*), Jin et al. [67] reported that the GtLCC1 gene plays a crucial role in lignin degradation. The *G. tsugae* isolates, having different GtLCC1 mutations, showed various ability levels to degrade lignin [67].

LCC genes in plants have different functions than those in fungi [60, 68, 69]. For example, the AtLCC genes in *Arabidopsis thaliana* [44, 46] plays an essential role in lignin polymerization, while those in *Lupinus albus* cv. Multolupa are involved in cell wall lignification [36]. Moreover, the LCC genes encodes polypeptides associated with cell wall structure in *Populus trichocarpa* [70]. Based on the nucleotide sequences available in the NCBI GenBank Database, the oil palm EgLCC24 gene had a high sequence identity to OsLCC24 from *Oryza sativa* [data not presented]. In rice, the function of the OsLCC gene product was associated with cell wall lignification [71]*.* Hence, the oil palm EgLCC24 gene evaluated in this study might have the same function in cell wall lignification in oil palm root tissues since oil palm LCC24 is expressed in roots.

Our findings also indicated that the increased expression of the oil palm EgLCC24 gene in resistant seedlings highlighted the possibility of higher gene regulation during *G. boninense* infection. On the other hand, oil palm EgLCC24 gene expression was lower in susceptible seedlings upon infection with *G. boninense*. One explanation for this is that the ability to suppress EgLCC24 gene expression may be one way to avoid *G. boninense* infection. Further studies of the function of the oil palm EgLCC24 gene associated with the responses to *G. boninense* infection should be carried out. Moreover, the interaction between EgLCC24 gene function in oil palm and *G. boninense* in lignin degradation may need further investigation.

Second, EgGSTU19 genes were highest expressed in response to basal stem rot (BSR) disease in susceptible oil palm seedling roots. The EgGSTU19 gene was highly expressed in the roots of susceptible seedlings infected with BSR disease, and the increase in expression was 0.72 times higher than that in uninoculated seedlings. The increased expression of the EgGSTU19 gene indicates a gene for susceptibility to *Ganoderma*. The AtGSTU19 protein interacts closely with cytosolic and peroxidase activity [41]. Furthermore, the AtGSTU19 gene involved in drought response of *A. thaliana* [72, 73].

Third, genes of EgROMT, EgWAKL5, and EgGLT3 were not different among the evaluated samples and were not induced by *G. boninense*. The EgROMT gene has a molecular function as a trans-resveratrol di-O-methyltransferase located in the cytoplasm, plastids, and mitochondrion [74]. The protein from EgROMT is classified to the stress response group [74], and CsROMT is involved in the biosynthesis of pterostilbene in the cucumber [75]. VvROMT is expressed by wounding 24 h after inoculation *Plasmopara viticola* pathogens causes downy mildew disease on grapevine (*Vitis vinifera*) [76].

The AtWAKL5 gene was strongly expressed in roots, young seedlings, flowers, and abscission zone and was not found in mature leaves or stems [43]. AtWAKL5 gene expression is also involved in a plant’s response to pathogen infection, mainly induced by wounding stress and SA [43]. The biological function of WAKLs families have been identified in several plants. The CsWAKL08 strongly regulates citrus bacterial cancer resistance through induced SA, methyl jasmonate acid, and PRX activities [77]. In the Arecanut palm (*Areca catechu* L.), the AcGLT3 gene was included in the 20 genes upregulated in seedlings with albinotic leaves compared with normal leaf seedlings. These genes were related to the development and response of plant hormones [78]. Finally, only one gene was identified as potential candidate for biomarkers against Ganoderma infection in oil palm, which is the EgLCC24 for selecting resistant seedlings. The molecular marker approach with these gene will widely encourage the acceleration of the oil palm breeding program regarding biotic stress from BSR disease in oil palm plantations.

In the early stages of infection, the plant does not detect the existence of pathogens. The detection activates genes involved in *Ganoderma* resistance response when the pathogen switches its nature to the necrotrophic phase. Evaluated genes are classified into 5 categories based on Pearson’s correlation analysis (Table 2), namely 1) receptor gene consisting of EgWAKL5 and EgMIK1; 2) genes involved in biosynthesis signals transduction compound such as EgOPR5 and EgACO1; 3) the response genes consisting of EgROMT, EgSOT12, EgLCC24, EgGLT3, and EgGSTU19; 4) one gene as trans-resveratrol di-O-methyltransferase-like (EgRNaseIII) predicted related to BSR infection. At the same time, two other genes are remained unknown (EgUnk1, EgUnk2).

The AtWAKL5 receptor gene is expressed in cells that experience wounding stress [43], but the EgMIK1 gene acts as a receptor gene on the plant's reproductive system. The AtMIK1 is a LURE receptor that was able to increase the ability of directed growth in the sexual reproductive system of plants [79, 80]. In addition, the AtMIK1 gene plays a functional role in ligand and receptor binding [81]. In the same family, AtMIK2 plays a role during root growth, development, and stress response [82] to the fungal pathogen of *Fusarium* spp. [83, 84]. Further, Van der Does et al. [84] stated that AtMIK2 controlled root growth and response to biotic stress of the pathogen *F. oxysporum* and abiotic stress of salinity tolerance.

Second, the EgOPR5 transduction signal gene plays a role in jasmonate biosynthesis [42] and ZmOPRs plays a role in hormone stress or pathogen infection [85]. The difference between PsOPR5 and other PsOPRs is that the PsOPR5 gene not responsive to the coronatin phytotoxin as jasmonic acid produced by the pathogen *Pseudomonas syringae* in peas [86]. On the other hand, OsOPR5 was significantly up-regulated in roots of *Meloidogyne graminicola* resistant rice plants [79]. The other gene, the EgACO1 involved in ethylene biosynthesis in the ﻿oil palm ripe fruit abscission [87] and ripening of strawberry fruits [88]. Ethylene is also known to play a role in plant transduction signals to biotic and abiotic stress [89].

Third, the resistance response genes to pathogenic infections. EgROMT is responsible for phytoalexin biosynthesis in *V. vinifera* by catalyzing resveratrol to pterostilbene [76]. EgSOT12 able to enhanced the inhibition of primary root growth by SA. Previous studies stated that the AtSOT12 gene plays a role in abiotic stress [40]. AtSOT12 gene expression is induced by abiotic stress and hormonal changes, especially in excessive salt and ABA conditions during seed germination in *A. thaliana* [40]. In addition, the AtSOT12 gene sulphonates SA [40].

LCCs gene acts as an essential enzyme in lignin biosynthesis [90]. Lignin is necessary as a physical defense of plants against pathogenic infections and is related to the development and response of plant hormones [91]. GSTU1s is a glutathione-s-transferase and relate to ROS [92]. The function of GSTUs is 1) stabilizing ROS at a certain level with a hypersensitivity mechanism and 2) reducing the ROS that appears first when there is a pathogen so that hypersensitive areas decrease in a later infection area [73, 92]. Based on the path of the mechanism of the gene expressions, the defense response to BSR's disease infection is a physical defense associated with lignification of the growth and development of oil palm seeds.

### Correlation among GDI, seedling growth, and development and seedling response

Correlation analysis was conducted to further evaluate the possible function of root lignin, PRX, and SA in the resistance response and to evaluate the effect of the resistance response to oil palm seedling growth and development. In this study, the GDI based on the performance of the seedling population was used as the measure for determining the resistance response [1, 23].

Goh et al. [93] showed that at two months after inoculation, there were positive correlations between the GDI and either the BD or the SH. However, five months after inoculation, the GDI was negatively correlated with either SH, BD, or leaf area. In another study, positive correlations were also observed between the genetic background and the early physiological responses of artificially inoculated oil palm seedlings [48]. Moreover, a positive correlation was observed between root lignin and root SA content. These data indicated that physical and biochemical defenses actively protects oil palm seedlings from *G. boninense* infection. On the other hand, PRX activity is negatively correlated with the GDI, which indicates that the higher the PRX activities are, the more resistant are the evaluated oil palm seedlings.

Furthermore, one month after the occurrence of internal symptoms of *G. boninense* infection, the root lignin content was negatively correlated with the observed oil palm seedling growth and development characteristics. The root SA content was also negatively correlated with BLN. Previous studies have also indicated that changes between biotrophic and necrotrophic stages of *G. boninense* can defeat oil palm seedlings [14, 91]. Therefore, more detailed investigations are needed to obtain complete pictures of oil palm roots and *G. boninense* interactions that lead to either resistance or susceptibility responses.

Based on the correlation analysis and expressed gene involved *Ganoderma* infection, we highlighted that the lignin content has a crucial role in oil palm response to *Ganoderma* infection. This statement was supported by growth and development of oil palm seedlings that has direct and indirect effect in the path analysis toward lignin content (Fig. 6) and the expression level of lignin responsible gene EgLCC24 were highly expressed in inoculated treatment (Fig. 5l).

The development of a new *G. boninense-*resistant oil palm variety is the focus of various groups of researchers. Data on the quantitative characteristics before and after infection symptoms appear post-inoculation are important in evaluating the pathogen effects. The ability to inhibit mycelial growth of *G. boninense* and activate the oil palm early defense system in seedling nurseries is essential for resistance against *G. boninense*. Other findings have also suggested physical defense mechanisms through lignin deposition [31, 33, 34, 61]. The activation of SA-associated defense mechanisms by SA has also been predicted in previous research [53, 84]. SA is also essential and a compound that activates systemic acquired resistance (SAR) [91] and many resistance genes [60].

## Conclusions

Oil palm seedlings infected with BSR disease did not affect growth and development, except for a decrease and damage to the weight of fresh roots. Among the evaluated seedling populations, the C03, C07, and C08 seedling populations were identified as resistant, while C01, C02, and C05 were susceptible to *G. boninense* infection*.* The C05 seedlings were the most susceptible populations, while the C08 seedlings were the most resistant. Physical defenses were induced in response in resistant oil palm seedlings from *G. boninense* infection. The root lignin, SA content, and PRX activities of oil palm seedlings associated with the resistance level were able to protect the oil palm seedlings from *G. boninense* infection. *Ganoderma boninense* infection affected the high expression of the EgLCC24 gene in resistant seedlings and the EgGSTU19 gene in susceptible seedlings. The EgLCC24 has an excellent opportunity as molecular biomarkers of oil palm BSR disease.

## Methods

### Plant materials

The evaluation was conducted at the IOPRI oil palm nursery at Marihat, Simalungun, North Sumatra, Indonesia. Oil palm seeds were germinated and planted in polybags (35 × 45 cm) containing 7.5 kg of top mineral soil and sand (1:1) mixtures for up to seven months. In this study, four different crosses with Dumpy *dura* × AVROS *pisifera* (C01, C02, C03, and C05) genetic background were obtained from the IOPRI. The four accesions were from the same AVROS *pisifera* pollen, but Dumpy *dura* is different (Tabel 1). Two crosses with Deli *dura* x La Mé *pisifera* (C07) and Deli *dura* x Yangambi *pisifera* (C08) genetic backgrounds from PT. Socfin Indonesia, North Sumatra were also included in the evaluation.

### Pathogen inoculum and inoculation

**Fig. 7:**
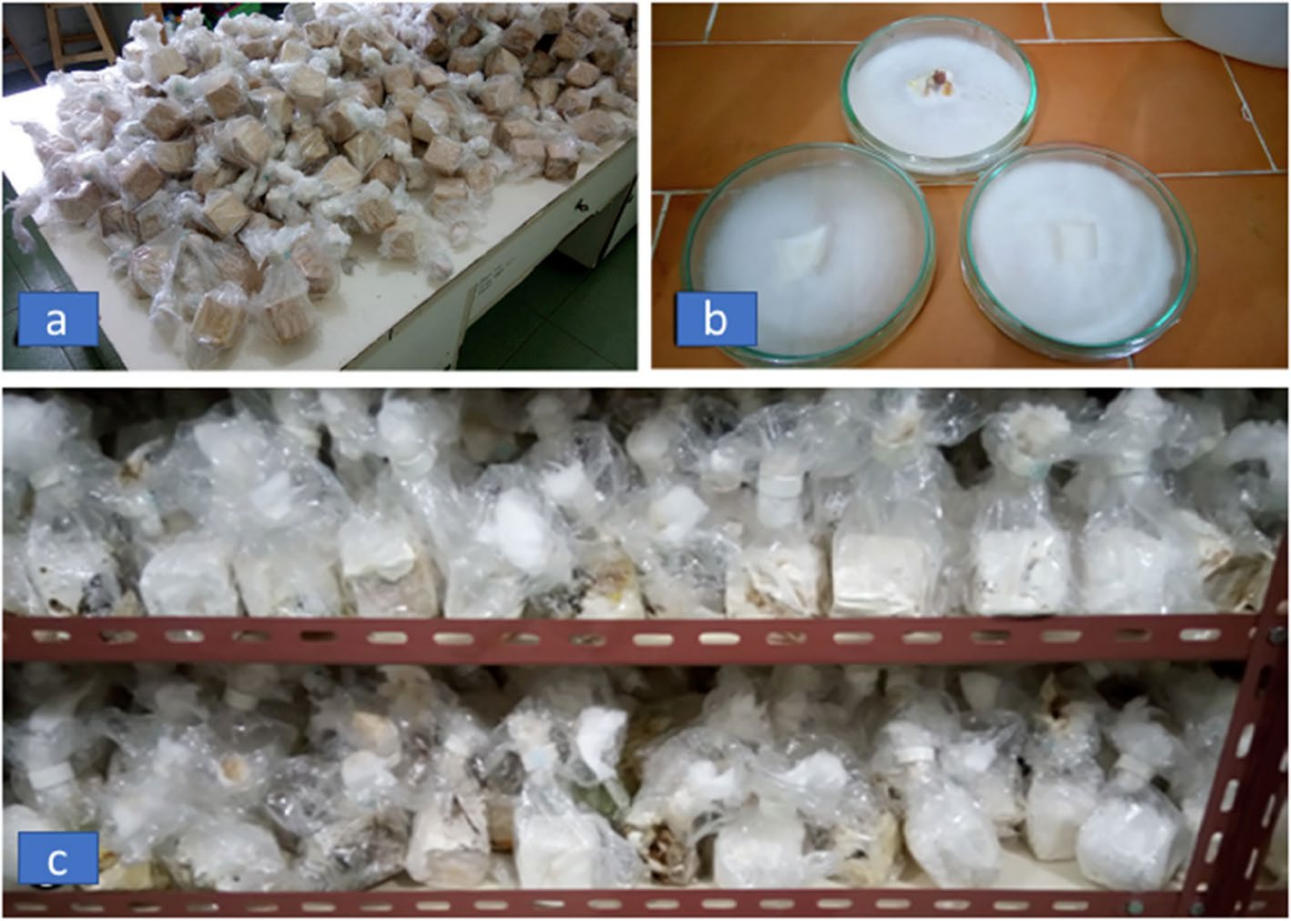
Preparation of inoculum sources of *Ganoderma boninense*. **a** Rubber woodblocks (RWB) before sterilization using an autoclave, (**b**) pure *G. boninense* isolate SSU008 as the source of inoculum, and (**c**) *G. boninense-*infected RWBs at two months after inoculation and ready for inoculation of oil palm seedlings

The *G. boninense* SSU008 isolate was grown following the IOPRI standard procedures in a 216 cm^3^ rubber woodblock (RWB, Fig. 7) [17]. The *G. boninense* SSU008 isolate originated from North Sumatra, and it was continually maintained by the Plant Protection Laboratory, IOPRI. Based on a previous report, the SSU008 isolate is consistently the most virulent *G. boninense* isolate available at the IOPRI [23]. Multiplication and cultivation of the *G. boninense* SSU008 isolate were performed following the procedures developed by Yanti and dan Susanto [94]. The rubber woodblocks (6 × 6 × 6 cm^3^) were bagged individually in a plastic bag and sterilized by autoclaving at 121 °C for 30 min (Fig. 7a). One agar block (1 cm^2^) of pure *G. boninense* isolate mycelia grown on PDA medium was inoculated into each of the RWBs (Fig. 7b), and the inoculated RWBs were incubated for two months at room temperature (25 °C) (Fig. 7c).

Germinated seeds of oil palm are planted around 4 months old (from harvest to germinate). The germinated seeds criteria are plumula length (prospective bole and leaves) and radicule (root candidates) measuring < 2 cm, healthy conditions, and seed size according to the Indonesian standard of oil palm seeds production. For inoculated oil palm seedlings, the *G. boninense-*infected RWBs were buried approximately 5 cm in the middle of the potting medium one day before transplanting the germinated seeds of oil palm [61]. Ganoderma infection occurs when the root of healthy germinated seeds grows and interact the source of the RWB inoculum in the media.

For control (healthy) seedlings, germinated oil palm seeds were planted on medium without *G. boninense-*infected RWBs. The growing oil palm seedlings were maintained for up to seven months. Some seedlings were sampled and evaluated monthly, starting at four months after transplant (before the first internal symptom occurrence) and up to seven months after transplantation (after the first internal symptom occurrence). The recorded data included the occurrence of internal infection symptoms of *G. boninense* infection, seedling growth, and biomass yield. Moreover, lignin content, peroxidase activities and SA content were also determined from the roots of each oil palm sample, both at 4 and 7 MAP.

### Response evaluation based on Ganoderma disease index

Visual observations and scoring were conducted for the external and internal symptoms in the shoots, roots, boles, and bifid leaves [48]. The recorded scores were used to calculate the DIs and the GDI [95]. Disease incidence was calculated as DI = [a/(a ​​ + b)] × 100%, in which [a] was the number of infected plants and [b] was the number of healthy plants. Subsequently, the DI and the score of symptoms were used to calculate the GDI [48, 95, 96]. The GDI was calculated based on the average DI for each seedling population divided by the average DI for the entire population. Grouping the evaluated seedlings into either resistant or susceptible against *G. boninense* infection was based on Turnbull et al. [1]. If the GDI value was lower than 100%, the seedlings were regarded as resistant. On the other hand, if the GDI was larger than 100%, the seedlings were regarded as susceptible.

### Growth and development of oil palm seedlings as affected by G. boninense infection

SH, BLN, BD, RL, and FRW were measured before (4 months after planting) and after the first symptoms of *G. boninense* infection (7 MAP). The observations were performed following the IOPRI standard procedures using 5 replicates with 8–10 seedlings per replicate with total 241 seedlings for inoculated plot and 244 seedlings for control plot.

### Lignin content, peroxidase activity, and salicylic acid content measurement

For LIGNIN, PRX, and SA content measurement, root samples were collected before (4 MAP) and after the first symptoms of *G. boninense* infection (7 MAP). Representative resistant and susceptible seedlings were sampled for analysis of LIGNIN, PRX, and SA content. A total of five seedlings were harvested and sampled and used for LIGNIN, PRX, and SA content measurements. After combining the five roots as one composite sample, the LIGNIN, PRX, and SA content measurements were conducted twice.

LIGNIN was determined by using the gravimetric method proposed by [95]. LIGNIN analysis was carried out at the IOPRI Services Unit, Medan and the Biochemistry and Molecular Biology Laboratory, Indonesian Research Institute for Biotechnology and Bioindustry (IRIBB), Bogor, Indonesia. PRX activities were measured using the spectrophotometry method developed by Saunders and McClure [96]. The root samples (± 1 g) were homogenized in a 0.1 M phosphate buffer at pH 6.5 (1–4 ml) and 4 °C. The homogenate was transferred into a 2 ml plastic tube and centrifuged at 6000 RPMs at 4 °C for 20 min. The supernatant was transferred into a new tube and ready for activity measurement. For peroxidase activity measurement, 1.5 ml of 0.05 M pyrogallol, 0.1 ml of enzyme extract, and 0.1 ml of 1% H_2_O_2_ were thoroughly mixed. The mixtures were read at 420 nm using a spectrophotometer. Analysis of SA content was carried out using high-performance liquid chromatography, as reported by Tenhaken and Rübel [97].

### Quantitative real-time PCR (qPCR) for involved genes

Twelve seedlings from four oil palm crosses progenies were used in this experiment. Resistant (C03, C07, and C08) and susceptible (C01) crosses with and without artificial inoculation were used in this experiment. C02 and C05 accessions were not included in the analysis because the quality of their RNA has been degraded and the results of PCR using the Actin gene did not show any amplicon in electrophoresis visualization. A sample calibrator was used on uninoculated susceptible seedlings as a unit sample to compare Log10 fold change values in the test samples [88]. Resistant and susceptible unit using three biological replicates and three technical replicates in the experimental unit and was tested on the 12 targeted genes.

Twelve targeted genes used in this experiment include cytosolic sulfotransferase 12-like (EgSOT12), MDIS1-interacting receptor-like kinase 1-like (EgMIK1), putative UPF0481 protein At3g02645 (EgUnk1), uncharacterized LOC105032389 (EgUnk1), 1-aminocyclopropane-1-carboxylate oxidase (EgACO1), ribonuclease 3 (EgRNaseIII), trans-resveratrol di-O-methyltransferase-like (EgROMT), crocetin glucosyltransferase 3-like (EgGLT3), wall-associated receptor kinase 5-like (EgWAKL5), glutathione S-transferase U19-like (EgGSTU19), putative 12-oxophytodienoate reductase 5 (EgOPR5), and Laccase-24 (EgLCC24). The target genes were normalized using the internal control Actin-101 (EgACT), which has been widely used and is stable as a reference gene in oil palm seedlings [97, 98].

The qPCR procedure also follows the guidelines for the Minimum Information for publication of Quantitative real-time PCR Experiments (MIQE) guidelines [99, 100] modified on biological replicates and technical replicates. Total RNA was isolated from 50 mg FRW of oil palm seedlings C01 (susceptible) and C03, C07, and C08 (resistant). Four seedlings were sampled for each population and used as biological replicates. The root tissues were collected seven months after planting. Total RNA was extracted using the Qiagen RNeasy® Plant Mini Kit by following the suggested manufacturer instructions. The DNase I kit from Sigma–Aldrich was used to eliminate DNA contaminants.

cDNA synthesis was performed using oligo(dT)20 primers and reverse transcriptase. Conventional PCR and real-time quantitative PCR (RT–qPCR) analysis were carried out as previously described [101]. Screening of 12 specific primers was performed to optimize the RT–qPCR of the target gene. Total twelve gene expression analyses were evaluated from 3 transcriptomic datasets from the leaves and roots of resistant (MTG-derived) and susceptible (Yangambi Socfindo-derived) oil palm. The accessions SRX4663593 (susceptible, leaf), SRX4663594 (resistant, root), and dan SRX4663595 (susceptible, root) were submitted by the Indonesian Research Institute for Biotechnology and Bioindustry. In addition, the selection of 12 targeted genes was based on a literature study on genes involved in the biological functions of plants stressed by biotic and abiotic stress [40, 43, 66] and preliminary analysis of differential gene expression in oil palm leaves and roots infected with *G. boninense*.

The constitutively expressed actin gene was used as the internal control for RT–qPCR analysis. The specific primers were forward primers and reverse primers, which are described in Table 5. The RT–qPCR reaction mixtures consisted of 5 μL SYBR Green, 0.625 μL each of forward and reverse primers, 3.75 μL NFW buffer, and 1 μL cDNA. The RT-q PCR procedures followed the methods developed by Faizah et al. [101] and Putranto et al. [97].

**Table 5:** List of primers used to determine the relative expression of genes in *Ganoderma* infection

No	Forward	Reverse	Gene description	Gene symbol	Chromosome
1	GATTGTGGCTTTTGTTGGAG	CTGAGGCTTCTGGCGTATTG	MDIS1-interacting receptor like kinase 1-like	LOC105051778	9
2	TACATTCCTGGCGGCTATCT	CGTTCTTTCCAGTCCTTTCC	putative UPF0481 protein At3g02645	LOC105032287	Un
3	CCTCGAATGCCCTATGTCTC	CCTCAAGGGATGTGCAATGT	uncharacterized LOC105032389	LOC105032389	Un
4	ACCATAGCTCCAGCAACCAC	CCATGCAACCTTGTTTCTCA	1-aminocyclopropane-1-carboxylate oxidase	LOC105035107	Un
5	CAGAGGGGATCAAAGCAACT	GAGATGTTGTGGGGCTGTTC	ribonuclease 3-like	LOC105038719	1
6	CCTAAGAAAGATGGCGGAAA	AGTCGCTAAAACCTGCAACA	*trans*-resveratrol di-O-methyltransferase-like	LOC105040967	3
7	AAAAGGCAAGGTGGGTGACT	TCATTCCACAAGACGAGCAA	cytosolic sulfotransferase 12-like	LOC105041070	3
8	TGGATGAGTTCGTGGAGATG	GTGCCCTATTCACGTTTTTG	crocetin glucosyltransferase 3-like	LOC105043120	4
9	AGAAGCAGTGGATGGTCAAA	ATTCGGATAAGTGCCTGGTG	wall-associated receptor kinase 5-like	LOC105044008	4
10	GACGACGGCAGTGAATAGGT	GGGTGGTGGAAGGGTAGTTT	laccase-24	LOC105046308	5
11	GCAAAAGGTGCATGGAGAG	AGAAAACACCCTGCTCGAAA	glutathione S-transferase U19-like	LOC105046783	6
12	TGCCGGAGGATACGATAGAG	GCAGTAGACCCGAGAAATGG	putative 12-oxophytodienoate reductase 5	LOC105051589	9

*Un* unknown chromosome location

### Data analysis

The experimental design was conducted in a randomized complete block design. The evaluated treatments included six oil palm seedling populations; each population was inoculated with *G. boninense* or left uninoculated (control plot). A total of 600 oil palm seeds were planted, 300 seeds were inoculated in polybags, and 300 were planted as a control (Supplementary Fig. 1). The experimental unit consisted of 12 seedlings, and it was replicated five times. Distribution of seedlings samples included: Eight to ten seedlings per replicate were used for observation 4 MAP, or the phase before symptoms appeared (Supplementary Fig. 2) until the age of 7 MAP for observation of SH, BLN, and BD. Five other seedlings per replicate were removed from polybags for observation of time and types of internal symptoms, RNA samples for all accessions except C02 and C05, RL, FRW, SA, PRX, and LIGNIN. At the end of the observation seven MAP, approximately 5–7 seedlings per replicate were observed again for parameters SH, BLN, BD, LR, FRW, SA, PRX, and LIGNIN. There was a reserve of 2 seeds per replication to prevent germinated seeds from growing. All data were evaluated for their normal distribution and fitness to analyze two-way variance (ANOVA) using Statistical Tool for Agricultural Research (STAR) program (http://bbi.irri.org/products). Where appropriate, mean comparisons were performed using the Tukeys's Honest Significant Difference (HSD) test α = 0.05 or t-test at p ≤ 0.05. Pearson correlation analysis was performed to associate the LIGNIN, PRX, SA content, and ​​the responses of GDI evaluated oil palm seedlings against *G. boninense* infection. Path analysis using R 4.0.5 version was used to calculate the direct and indirect effects of the growth and biomass yield of *G. boninense*-infected oil palm seedlings 4 months after planting.

The qPCR calculation followed the Ct comparative method [102, 103], modified in Box 5 and due to the limited samples and technical research in the laboratory. Modifications and previous experiments were performed on three cDNA samples (sample 28, sample 40, and sample 55), using only Actin-101 primers with dilution levels of 5x (100 ng/µL), 10x (50 ng/µL), 25x (25 ng/µL), and 50x (10 ng/µL). The three cDNA samples mentioned, sample 28, sample 40, and sample 55, were samples from accessions C01 and C08 for determining the optimal cDNA concentration, which was 50 ng/µL. These results were used to calculate the PCR efficiency only for the Actin gene and as a basis for the concentration of cDNA samples in the targeted gene. Supplementary Fig. 3 shows a slope value of − 3.45 with an R2 value of more than 0.99. PCR efficiency [104] was calculated using the Formula E = (10 − 1/slope-1) × 100 and showed an amplification factor of 1.95 with an efficiency value of 94.92%. Assuming that the Actin reference gene was relatively stable in PCR amplification and there was one peak in each target gene for the analyzed samples (Supplementary Fig. 4), the concentration of cDNA used was the same for each primer. Determination of the primary effectiveness of the target gene was based on melt curve analysis with one Tm°C peak per target gene. The purpose of this dilution was not to obtain PCR efficiency for all targeted genes. However, it acquired uniform cDNA concentrations and the appearance of Ct values in the qPCR analysis. In addition, it minimized error factors and human errors during technical research. After obtaining the optimal concentration for all cDNA samples, an experimental matrix was made with three replications per sample for each targeted gene.

The expression values were calculated using the Step One Plus Real-Time PCR System and Step One Software v2.3 (Applied Biosystems, UK), as suggested by the manufacturers. The transcript accumulation of the 12 targeted genes or Cт of targeted genes was normalized using the accumulated transcript of the endogenous Actin-101 gene of oil palm (△Cт). Furthermore, △Ct of the test samples was normalized to the △Ct of the calibrator sample (△△Cт). The last fold difference was calculated in an experiment (2^-△△Cт). The difference of expression level for each gene across different resistant and susceptible within inoculated and control group were analyzed using Least Significant Difference (LSD) test was performed in STAR program. The relative gene expression level was visualized using clustered column chart.

## Supplementary Information

**Additional file 1:** **Figure 1.** Field design for *Ganoderma* infection experiments. White polybags for control plot, while red polybags for *Ganoderma* *boninense* infection. **Figure 2.** Performance of oil palm seedlings at 4 months after planting. Red polybags were infected *Ganoderma* and white polybags were control plot in the observations of growth and development of oil palm seedlings. **Figure 3.** PCR efficiency of Actin-101 gene. **Figure 4.** Melt curve analysis of the 12 targeted genes.

## Data Availability

The full-length transcriptome data were submitted to the public repository SRA database and are available at the following links: https://www.ncbi.nlm.nih.gov/sra/SRX4663593[accn], https://www.ncbi.nlm.nih.gov/sra/SRX4663594[accn], and https://www.ncbi.nlm.nih.gov/sra/SRX4663595[accn]. The public access to the databases is open.

## Abbreviations

G. *boninense*: *Ganoderma* *boninense*
BSR: Basal stem rot
IOPRI: Indonesian Oil Palm Research Institute
PRX: Peoxidase
SA: Salicylic acid
GDI: *Ganoderma* Disease index
DI: Disease incidence
RWB: Rubber wood block
SH: Seedling height
RL: Root length
BLN: Bifid leaf number
BD: Bole diameter
FRW: Fresh root weight
r: Pearson correlation coefficient
LIGNIN: Root lignin content
CWDEs: Cell wall degrading enzymes
MnP: Manganese peroxidase
LiP: Lignin peroxidase
qPCR: Quantitative real-time PCR
SOT12: Cytosolic sulfotransferase 12-like
MIK1: MDIS1-interacting receptor-like kinase 1-like
At3g02645: Putative UPF0481 protein At3g02645
LOC105032389: Uncharacterized LOC105032389
ACO1: 1-Aminocyclopropane-1-carboxylate oxidase
RNaseIII: Ribonuclease 3
ROMT: Trans-resveratrol di-O-methyltransferase-like
GLT3: Crocetin glucosyltransferase 3-like
WAKL5: Wall-associated receptor kinase 5-like
GSTU19: Glutathione S-transferase U19-like
OPR5: Putative 12-oxophytodienoate reductase 5
LCC24: Laccase-24
