# Supplementary material for: Defense response changes in roots of oil palm (Elaeis guineensis Jacq.) seedlings after internal symptoms of Ganoderma boninense Pat. infection
Source: BMC Plant Biol. 2022 Mar 24;22:139. doi: 10.1186/s12870-022-03493-0 (PMC8944027; doi:10.1186/s12870-022-03493-0)
Supplement: Supplementary file 1 — Additional file 1: Figure 1. Field design for Ganoderma infection experiments. White polybags for control plot, while red polybags for Ganoderma boninense infection. Figure 2. Performance of oil palm seedlings at 4 months after planting. Red polybags were infected Ganoderma and white polybags were control plot in the observations of growth and development of oil palm seedlings. Figure 3. PCR efficiency of Actin-101 gene. Figure 4. Melt curve analysis of the 12 targeted genes. [file 12870_2022_3493_MOESM1_ESM.docx]

Supplementary


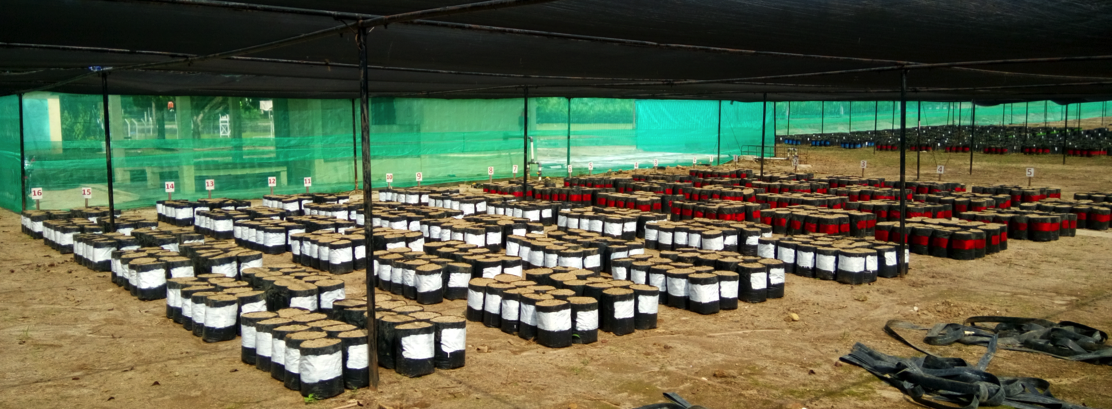


Supplementary Figure 1. Field design for *Ganoderma* infection experiments. White polybags for control plot, while red polybags for *Ganoderma boninense* infection.


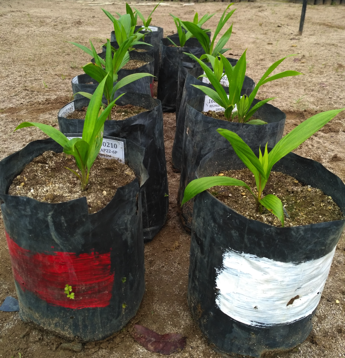

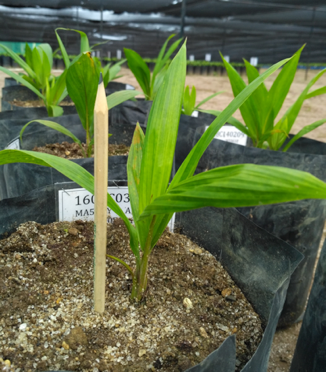


Supplementary Figure 2. Performance of oil palm seedlings at 4 months after planting. Red polybags were infected *Ganoderma* and white polybags were control plot in the observations of growth and development of oil palm seedlings.

Supplementary Figure 3. PCR efficiency of Actin-101 gene.


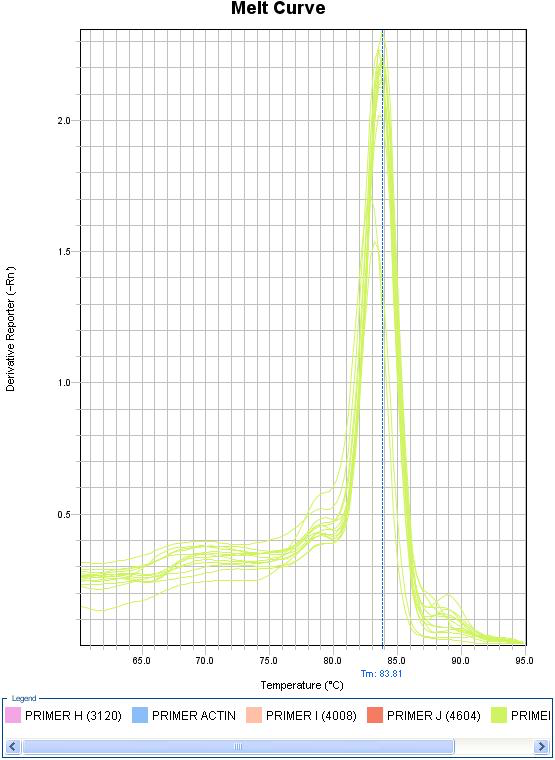

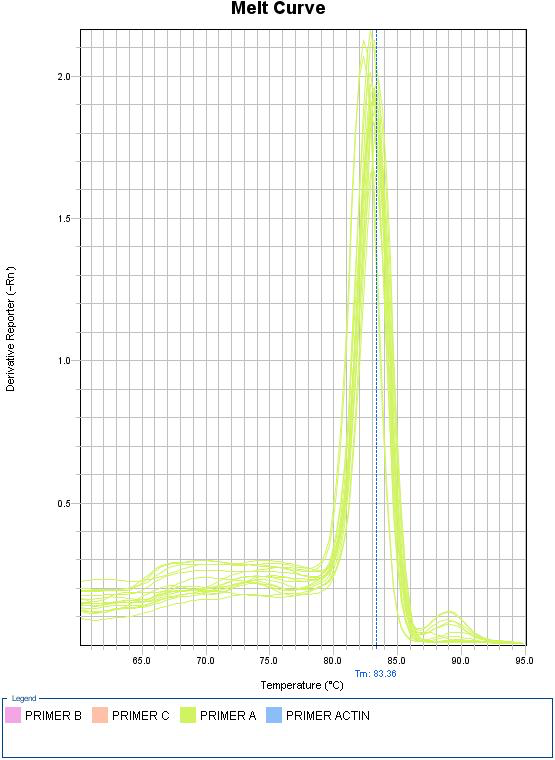

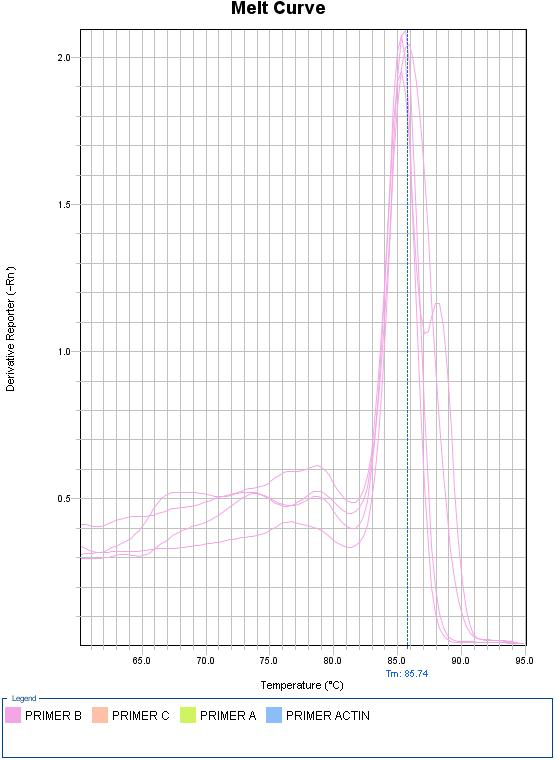


EgSOT12 EgMIK1 EgUnk1


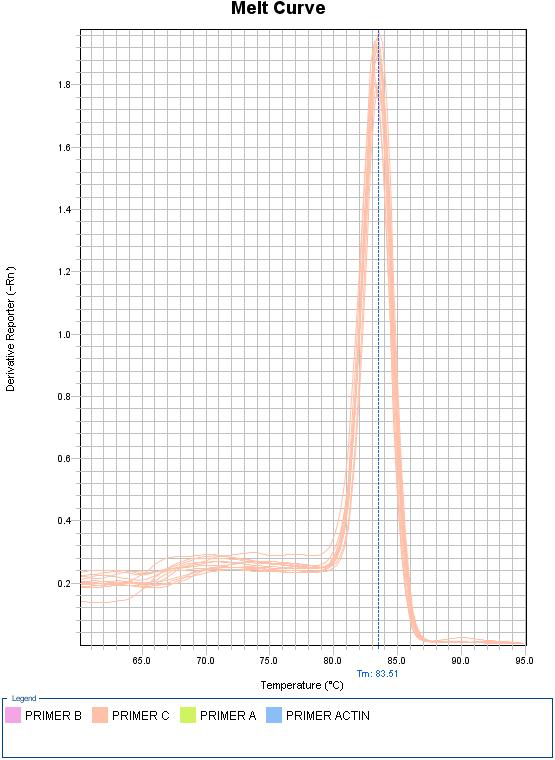

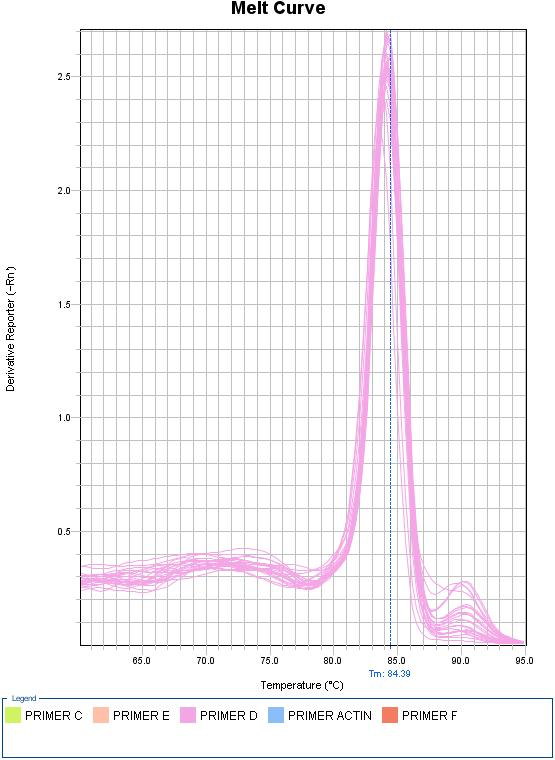

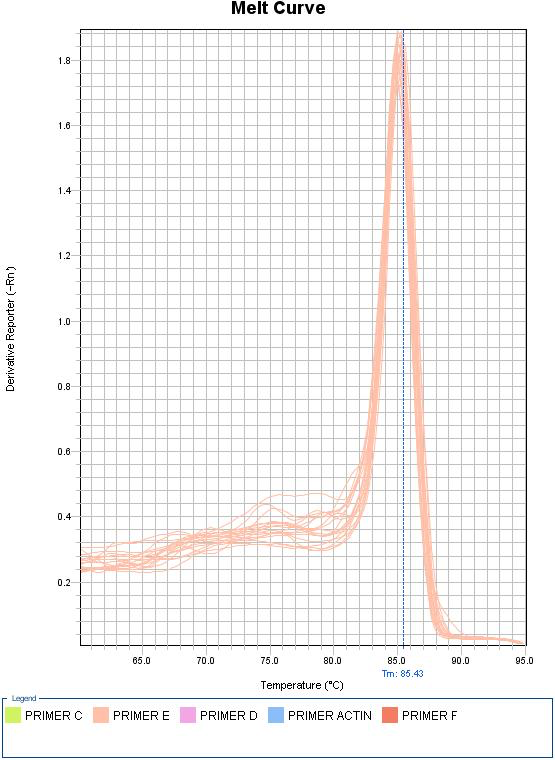


EgUnk2 EgACO1 EgRNaseIII


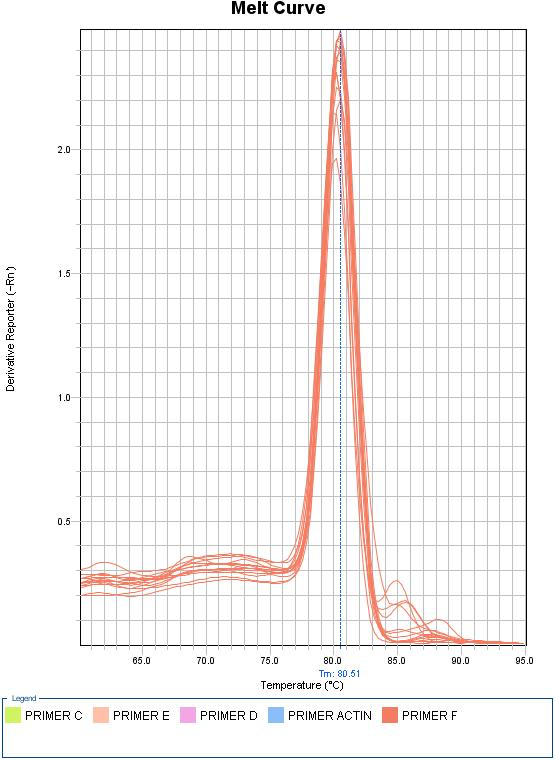

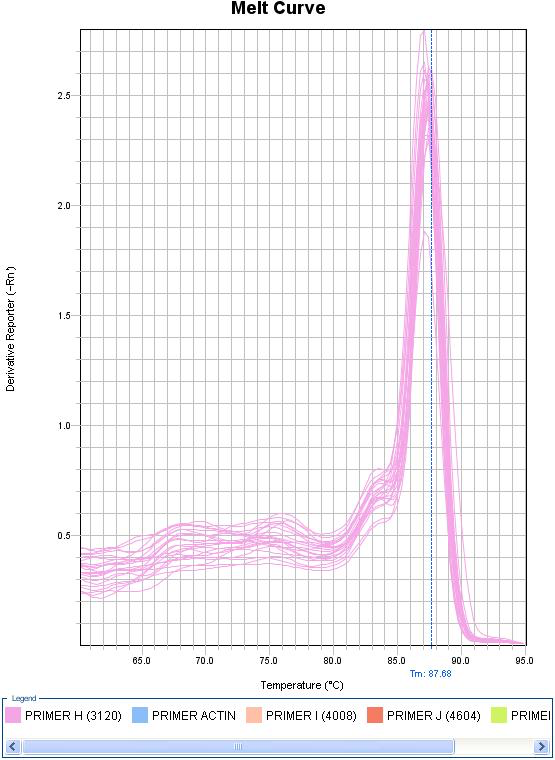

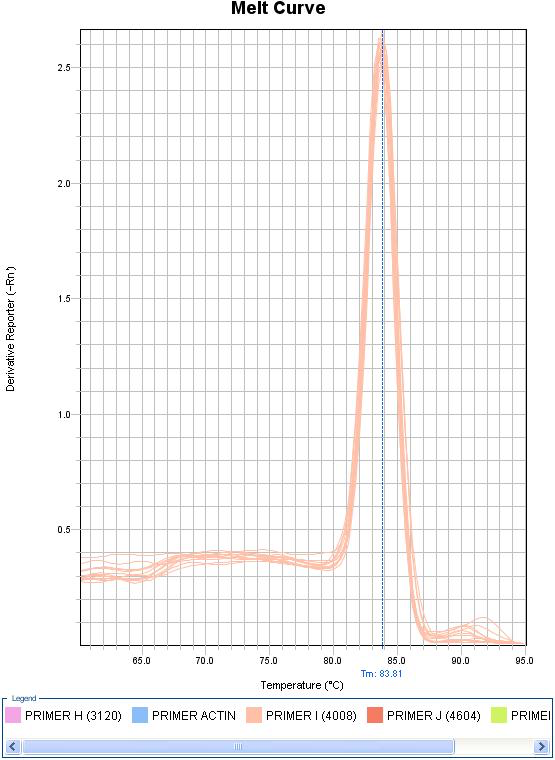


EgROMT EgGLT3 EgWAKL5


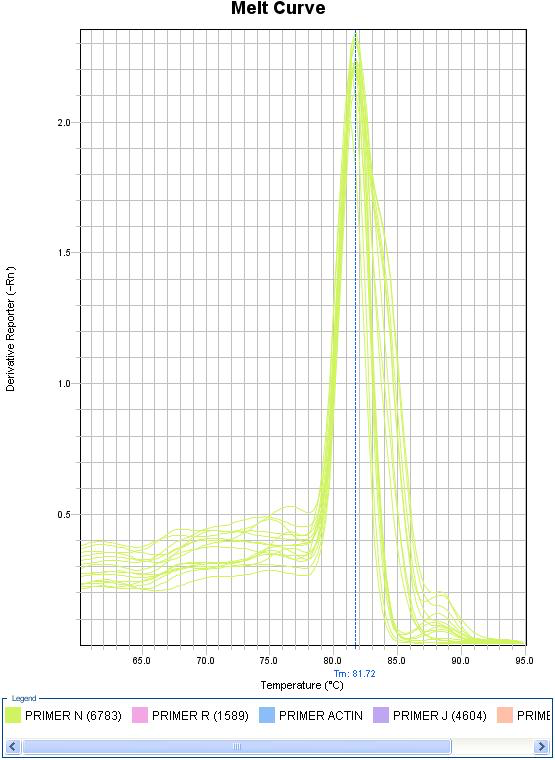

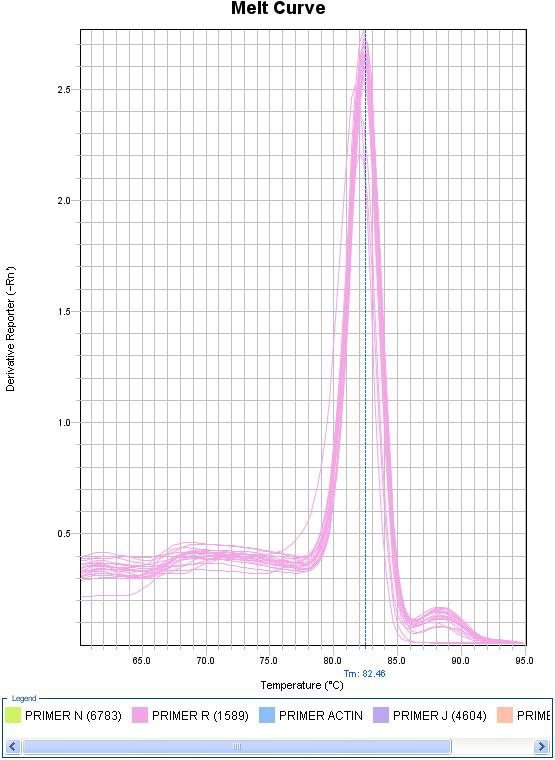

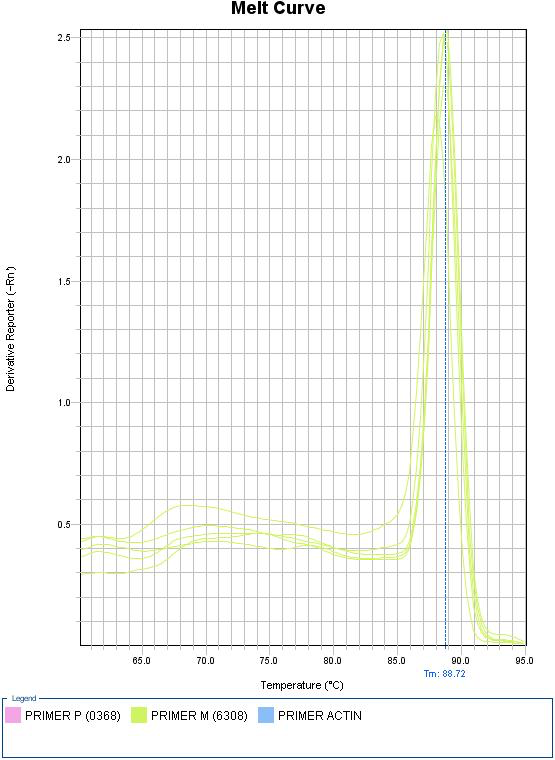


EgGSTU19 EgOPR5 EgLCC24

Figure 4. Melt curve analysis of the 12 targeted genes.
